# Rising colorectal cancer burden attributable to high body mass index in China from 1990 to 2021: a comprehensive analysis using the global burden of disease study

**DOI:** 10.3389/fendo.2025.1509497

**Published:** 2025-05-15

**Authors:** Xiamei Chen, Bijuan Chen, Sufang Ye, Hui Lin, Chunkang Yang, Zhouwei Zhan

**Affiliations:** ^1^ Department of Operation, Clinical Oncology School of Fujian Medical University, Fujian Cancer Hospital, Fuzhou, Fujian, China; ^2^ Department of Radiation Oncology, Clinical Oncology School of Fujian Medical University, Fujian Cancer Hospital, Fuzhou, Fujian, China; ^3^ Department of Medical Oncology, Clinical Oncology School of Fujian Medical University, Fujian Cancer Hospital, Fuzhou, Fujian, China; ^4^ Department of Gastrointestinal Surgery, Clinical Oncology School of Fujian Medical University, Fujian Cancer Hospital, Fuzhou, Fujian, China

**Keywords:** colorectal cancer, high body mass index, epidemiological change, age-period-cohort analysis, decomposition analysis, China

## Abstract

**Background:**

Colorectal cancer (CRC) attributable to high body mass index (BMI) has become a significant public health issue in China. This study analyzes the burden of CRC attributable to high BMI from 1990 to 2021, exploring trends in mortality, disability-adjusted life years (DALYs), years lived with disability (YLDs), and years of life lost (YLLs).

**Methods:**

Data were obtained from the Global Burden of Disease Study (GBD) database. Age- and sex-specific mortality, DALYs, YLDs, and YLLs rates were analyzed for 2021, and temporal trends were examined from 1990 to 2021. Joinpoint and age-period-cohort (APC) analyses were conducted to identify shifts in burden and contributing factors. Decomposition analysis was applied to assess the impact of aging, population growth, and epidemiological changes.

**Results:**

In 2021, 19,418 CRC deaths were attributable to high BMI in China, with a higher burden observed in males. The total DALYs reached 507,316, and YLLs accounted for 482,925. From 1990 to 2021, age-standardized mortality rates increased by 2.43-fold, while DALYs and YLLs increased by 2.33-fold and 2.24-fold, respectively. The most dramatic increase was seen in YLDs, with a 5.17-fold rise. Epidemiological changes contributed most to the increases in deaths and DALYs, followed by aging, while population growth had the least impact.

**Conclusions:**

The burden of CRC attributable to high BMI in China has grown significantly over the past three decades, with males disproportionately affected. Aging and epidemiological changes are the main drivers of this trend, underscoring the need for targeted interventions to reduce the CRC burden.

## Introduction

Colorectal cancer (CRC) is a leading cause of cancer-related mortality worldwide, with incidence rising rapidly in developing countries, particularly in China (1). Over the past three decades, CRC has emerged as a major public health challenge in China, with substantial increases in both incidence and mortality. Recent studies indicate that CRC is one of the fastest-growing cancer burdens in the country, disproportionately affecting males and younger individuals (2, 3). High body mass index (BMI) has been identified as a significant risk factor for CRC, contributing to both its development and progression (4–7). According to the World Health Organization (WHO), obesity-related cancers account for a substantial portion of the global cancer burden, making high BMI a critical modifiable risk factor with significant public health implications (8).

A large cohort study by Carr et al. highlighted that higher BMI is associated with specific CRC subtypes, including microsatellite instability-high (MSI-high) tumors and BRAF-mutated CRCs (9). Excess weight is associated with an increased risk of CRC, poorer prognosis, and higher mortality rates (10, 11). In China, rapid socioeconomic and lifestyle changes (e.g., dietary shifts, urbanization, reduced physical activity, especially in urban areas) have led to rising obesity rates (12), making it essential to assess the extent to which BMI contributes to the CRC burden. Despite these established associations, limited research has examined long-term trends in CRC burden attributable to high BMI in China. A comprehensive analysis of the contribution of high BMI to CRC burden is urgently needed. This study aims to fill this gap by providing an in-depth analysis of the burden of CRC attributable to high BMI in China from 1990 to 2021, focusing on temporal trends in mortality, disability-adjusted life years (DALYs), years lived with disability (YLDs), and years of life lost (YLLs).

The Global Burden of Disease (GBD) Study was selected as the data source due to its standardized methodology, integrating national surveys, cancer registries, and hospital records to provide comparable estimates of CRC burden attributable to high BMI in China. While prior studies have linked obesity to CRC risk, most relied on individual-level cohorts or regional datasets, lacking a nationwide, long-term perspective. This study uniquely examines three decades (1990-2021) of national-level data, providing a comprehensive assessment of temporal trends. Given China’s rapid urbanization, dietary shifts, and rising obesity prevalence, understanding the impact of these sociocultural changes on CRC burden is critical. This study is the first to apply Joinpoint regression and age-period-cohort (APC) analysis to identify key inflection points and generational effects in CRC burden related to high BMI. Additionally, decomposition analysis quantifies the contributions of aging, population growth, and epidemiological factors, distinguishing this study from prior research and informing targeted prevention strategies for CRC control in China.

## Material and methods

### Study design and data source

This study was conducted in accordance with ethical guidelines and principles for research involving publicly available data. Ethical approval and informed consent were not required, as the study utilized secondary data from the GBD 2021 database, which is publicly accessible and de-identified. The GBD 2021 database was used to analyze the burden of CRC attributable to high BMI in China from 1990 to 2021. The GBD database provides comprehensive estimates for 371 diseases and injuries across 204 countries and territories, integrating data from multiple sources, including population-based cancer registries, national health surveys, hospital discharge records, and vital registration systems. Data quality is assessed using standardized processing methods to ensure consistency and comparability across different regions and time periods (13). Statistical modeling within the GBD study involves a series of estimation procedures, including the Cause of Death Ensemble Model (CODEm) for mortality estimation and the Bayesian meta-regression tool DisMod-MR 2.1 for prevalence and incidence estimation. CODEm employs multiple predictive covariates and statistical algorithms to generate robust mortality estimates, while DisMod-MR 2.1 synthesizes heterogeneous data sources using Bayesian inference to adjust for inconsistencies and biases. Estimates are refined through predictive validity tests and cross-validation techniques to enhance accuracy. To account for uncertainty in data sources and modeling processes, all GBD estimates are reported with 95% uncertainty intervals (UIs), derived using a Monte Carlo simulation approach with 1,000 draws (14, 15). Data on age-standardized mortality rates (ASMR), DALYs, YLDs, and YLLs were extracted from the GBD Study 2021 database, accessed on September 12, 2024, using the GBD Results Tool (http://ghdx.healthdata.org/gbd-results-tool).

### Definitions

The epidemiologic data for CRC attributable to high BMI in the GBD database were estimated using a comparative risk assessment (CRA) framework. This method calculates the population-attributable fraction (PAF) by integrating BMI exposure distributions, relative risks from large-scale epidemiological studies, and a theoretical minimum risk exposure distribution (TMRED). The PAF is then applied to CRC mortality and morbidity estimates, derived from the Cause of CODEm and DisMod-MR 2.1, to estimate the burden of CRC attributable to high BMI in terms of mortality, DALYs, YLDs, and YLLs. All estimates include 95% UIs generated through Monte Carlo simulations (16, 17). For CRC, the GBD 2021 study used disease classification based on the International Classification of Diseases (ICD) codes, which are detailed in **Supplementary Table 1**. High BMI was defined differently for adults and children. For adults (aged 20 years and older), high BMI was defined as a BMI greater than 20-23 kg/m^2^, depending on the specific risk threshold used in different regions. For children (aged 2-19 years), high BMI was defined as being overweight or obesity, based on the standards provided by the International Obesity Task Force (IOTF). These definitions were used throughout the analysis to assess the burden of CRC attributable to high BMI across different age groups. Definition of high BMI can be found in https://www.healthdata.org/research-analysis/diseases-injuries-risks/factsheets.

### Joinpoint regression analysis

Joinpoint regression analysis was conducted to detect significant changes in the temporal trends of age-standardized rates of CRC burden attributable to high BMI. This method segments the time-series data into distinct linear trends, estimating the average annual percentage change (AAPC) for each segment to characterize variations over time. The analysis was performed using the Joinpoint Regression Program (version 5.2.0; National Cancer Institute, USA) (18, 19). Breakpoints (joinpoints) were identified where statistically significant shifts in trend slopes occurred, indicating periods of accelerated or decelerated changes. The optimal number of joinpoints was selected using the Bayesian Information Criterion (BIC), ensuring model parsimony while maintaining goodness of fit. Statistical significance was assessed using a Monte Carlo permutation test, with a *P*-value <0.05 considered significant. A trend was deemed significant if the 95% confidence interval (CI) of the AAPC did not include zero. This approach provides a nuanced understanding of the shifts in CRC burden trends attributable to high BMI over the study period.

### Age-period-cohort analysis

An APC model was employed to evaluate the independent effects of age, period, and birth cohort on CRC mortality and DALYs attributable to high BMI while accounting for their inherent linear dependency (20, 21). Data for mortality and DALYs rates were extracted from the GBD 2021 database, categorized into 5-year age groups spanning 1990 to 2021, along with population estimates for each year (https://ghdx.healthdata.org/record/ihme-data/global-population-forecasts-2017-2100). In the GBD dataset, individuals under 5 years and over 95 years were grouped together. For the APC model, age groups were categorized as 0-4, 5-9, 10-14, up to 95-100, with “0” representing the under-five category. Mortality and DALY rates were calculated for 5-year periods (e.g., 1992-1996, 1997-2001, etc.), ensuring robust cohort analysis. The APC analysis was conducted using the apc.fit function in the Epi package in R (version 4.3.1), applying a logarithmic transformation to incidence rates to normalize distributions and stabilize variance. The relative risks associated with age, period, and cohort effects were estimated for distinct age groups, time periods, and birth cohorts. Model fit was assessed using the Akaike Information Criterion (AIC), and residual analyses were performed to evaluate adequacy and ensure appropriate model specification. These refinements allow for a precise decomposition of CRC burden trends related to high BMI across different generations and time periods.

### Decomposition analysis

To assess the contributions of population growth, aging, and epidemiological changes to changes in CRC mortality and DALYs attributable to high BMI, a decomposition analysis was conducted. This analytical approach disaggregates the overall change in disease burden into three key components (population growth, aging, and epidemiological change) allowing for a detailed examination of the primary factors driving the observed trends (15). The decomposition analysis was performed using standard demographic decomposition techniques, which quantify the proportion of changes in disease burden attributable to shifts in population size, age structure, and risk exposure. This method has been widely applied in disease burden studies to evaluate long-term trends. Estimates were derived separately for both sexes and for the total population to capture sex-specific variations in CRC burden attributable to high BMI.

### Ethical considerations

As this study utilized publicly available data from the GBD 2021 database, ethical approval and informed consent were not required. All methods were carried out in accordance with relevant guidelines and regulations for the use of public health data.

## Results

### Burden of CRC attributable to high BMI in 2021

In 2021, CRC attributable to high BMI posed a significant disease burden in China, with 19,418 deaths reported across all age groups. Males exhibited a higher mortality burden than females, with 11,181 deaths compared to 8,236 deaths, respectively. The ASMR was 0.94 per 100,000 people, with males (1.18 per 100,000) facing a greater risk than females (0.75 per 100,000). The DALYs reached 507,316, with males contributing 304,946 DALYs and females 202,371 DALYs. The age-standardized DALY rate was 24.22 per 100,000, with males (30.29 per 100,000) again experiencing a higher burden than females (18.49 per 100,000). YLDs accounted for a smaller portion of the burden, with an overall 24,391 YLDs, while YLLs contributed the majority, totaling 482,925 YLLs. The age-standardized YLL rate was 23.08 per 100,000, with males experiencing a substantially higher rate (28.9 per 100,000) than females (17.57 per 100,000) (**Table 1**).

**Table 1:** All-age cases and age-standardized deaths, DALYs, YLDs, and YLLs rates in 2021 for CRC attributable to high BMI in China.

Measure	All-ages cases			Age-standardized rates per 100,000 people		
	Total	Male	Female	Total	Male	Female
Deaths	19418 (8053, 32452)	11181 (4295, 19530)	8236 (3262, 14370)	0.94 (0.39, 1.58)	1.18 (0.45, 2.04)	0.75 (0.3, 1.31)
DALYs	507316 (209264, 853770)	304946 (118365, 530717)	202371 (80646, 353994)	24.22 (9.98, 40.69)	30.29 (11.79, 52.57)	18.49 (7.34, 32.36)
YLDs	24391 (9244, 41692)	14254 (5216, 25497)	10137 (3971, 17762)	1.14 (0.43, 1.96)	1.38 (0.51, 2.45)	0.91 (0.36, 1.6)
YLLs	482925 (200365, 815811)	290691 (113121, 507630)	192234 (76258, 337437)	23.08 (9.56, 38.92)	28.9 (11.28, 50.34)	17.57 (6.95, 30.84)

Values in parentheses indicate 95% uncertainty intervals (UIs), estimated using Monte Carlo simulations. Abbreviations: DALYs, disability-adjusted life-years; YLDs, years lived with disability; YLLs, years of life lost; CRC, colorectal cancer; BMI, body mass index.

### Age- and sex-specific burden of CRC attributable to high BMI in China, 2021

**Figure 1:**
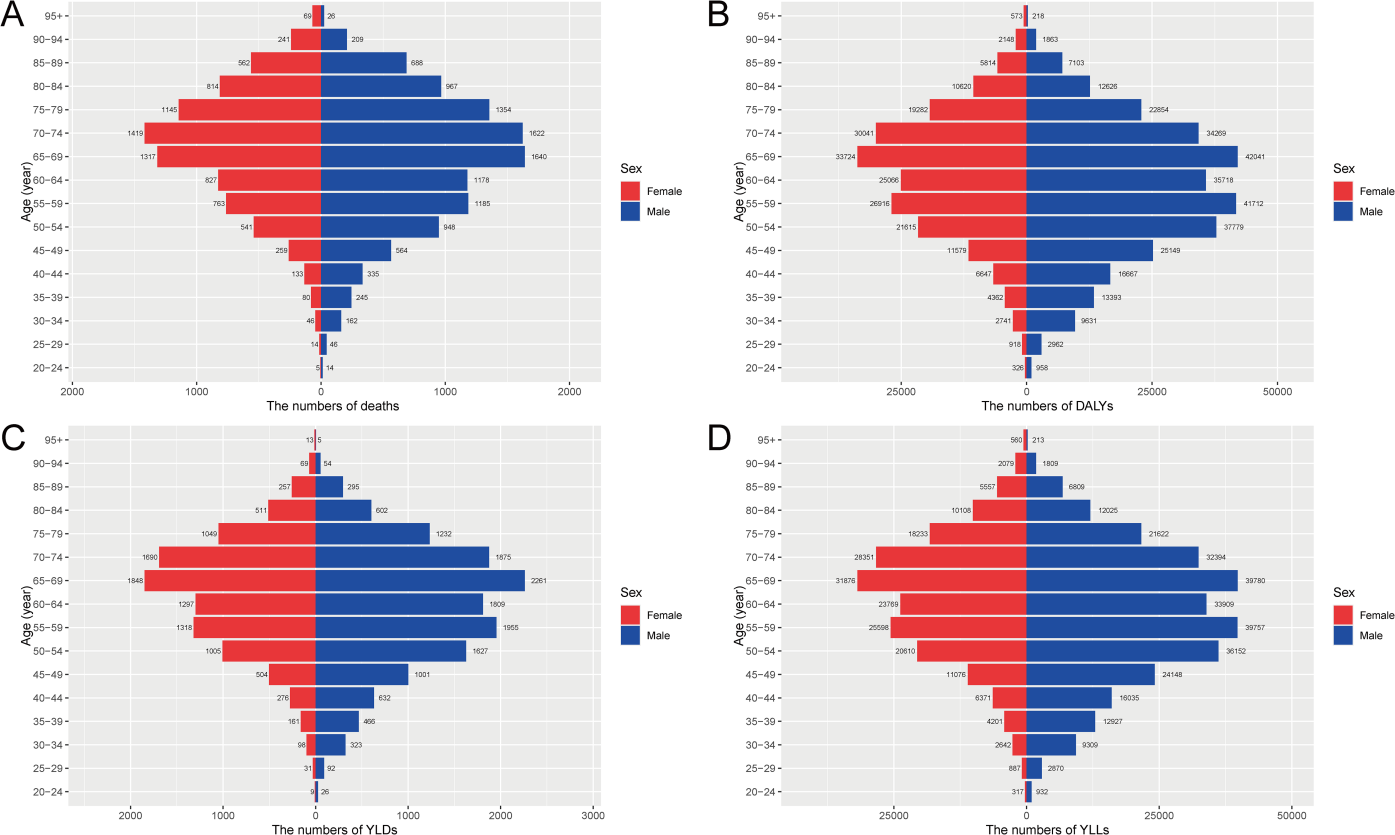
Age- and sex-specific numbers of deaths, DALYs, YLDs, and YLLs for CRC attributable to high BMI in China, 2021. **(A)** The numbers of deaths by age and sex. **(B)** The numbers of DALYs by age and sex. **(C)** The numbers of YLDs by age and sex. **(D)** The numbers of YLLs by age and sex. DALYs, disability-adjusted life years; YLDs, years lived with disability; YLLs, years of life lost; CRC, colorectal cancer; BMI, body mass index.

**Figure 2:**
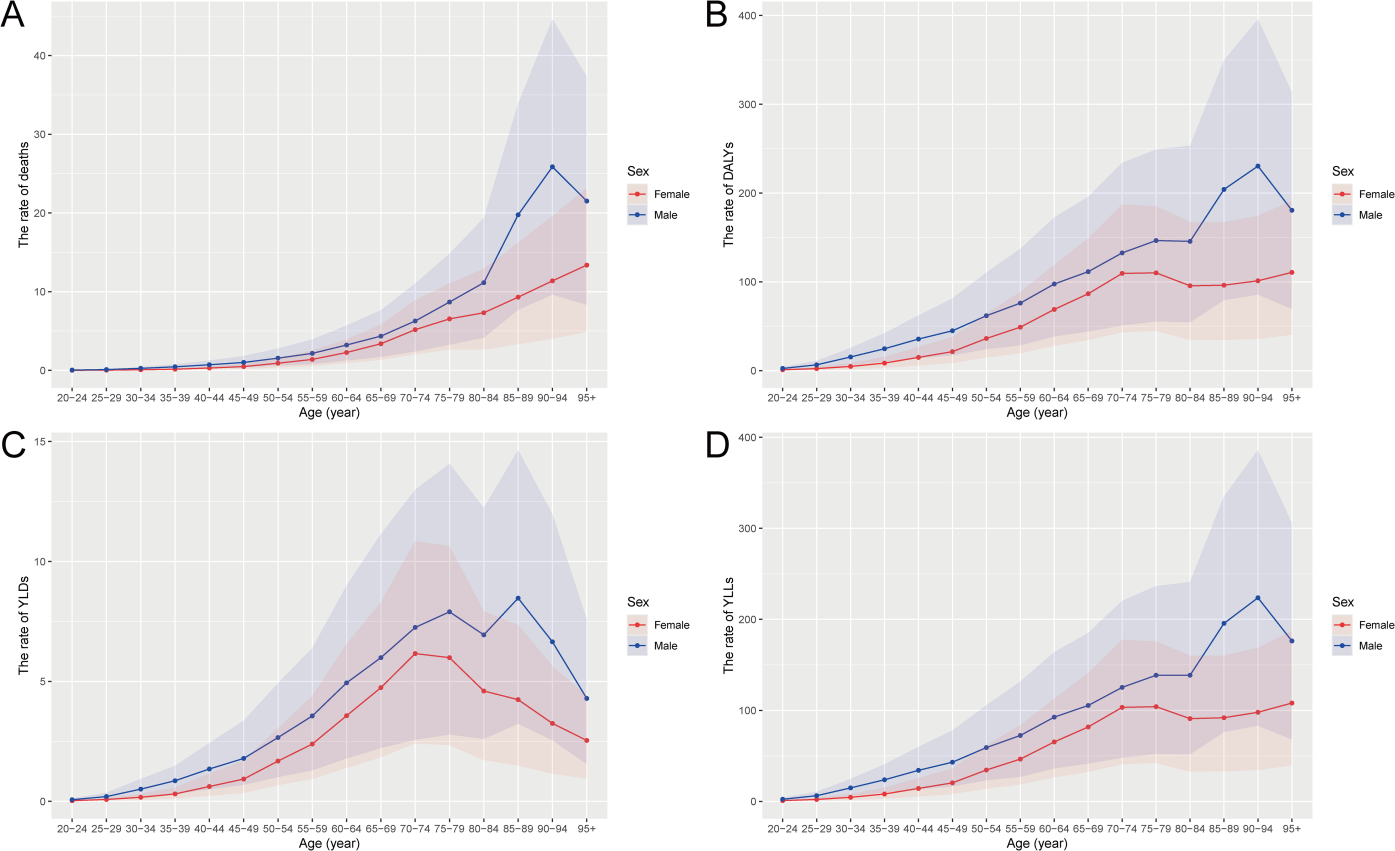
Age- and sex-specific rates of deaths, DALYs, YLDs, and YLLs for CRC attributable to high BMI in China, 2021. **(A)** The rate of deaths per 100,000 people by age and sex. **(B)** The rate of DALYs per 100,000 people by age and sex. **(C)** The rate of YLDs per 100,000 people by age and sex. **(D)** The rate of YLLs per 100,000 people by age and sex. DALYs, disability-adjusted life years; YLDs, years lived with disability; YLLs, years of life lost; CRC, colorectal cancer; BMI, body mass index.

In 2021, the burden of CRC attributable to high BMI showed marked variations by age and sex in China. The absolute numbers of deaths, DALYs, YLDs, and YLLs progressively increased with age, peaking in the 65-79-year age groups. Notably, males consistently exhibited higher absolute numbers across all metrics compared to females, with the peak occurring around the 70-74-year age group for both sexes (**Figure 1**). When considering rates per 100,000 people, age-specific rates of deaths, DALYs, YLLs, and YLDs increased dramatically in older age groups, particularly after age 50, reaching the highest levels among those aged 90 years and older. Again, males demonstrated significantly higher rates than females across all age groups, with particularly steep increases after age 65, highlighting the substantially greater risk for elderly males (**Figure 2**)​.

### Temporal trends in CRC burden attributable to high BMI from 1990 to 2021

**Figure 3:**
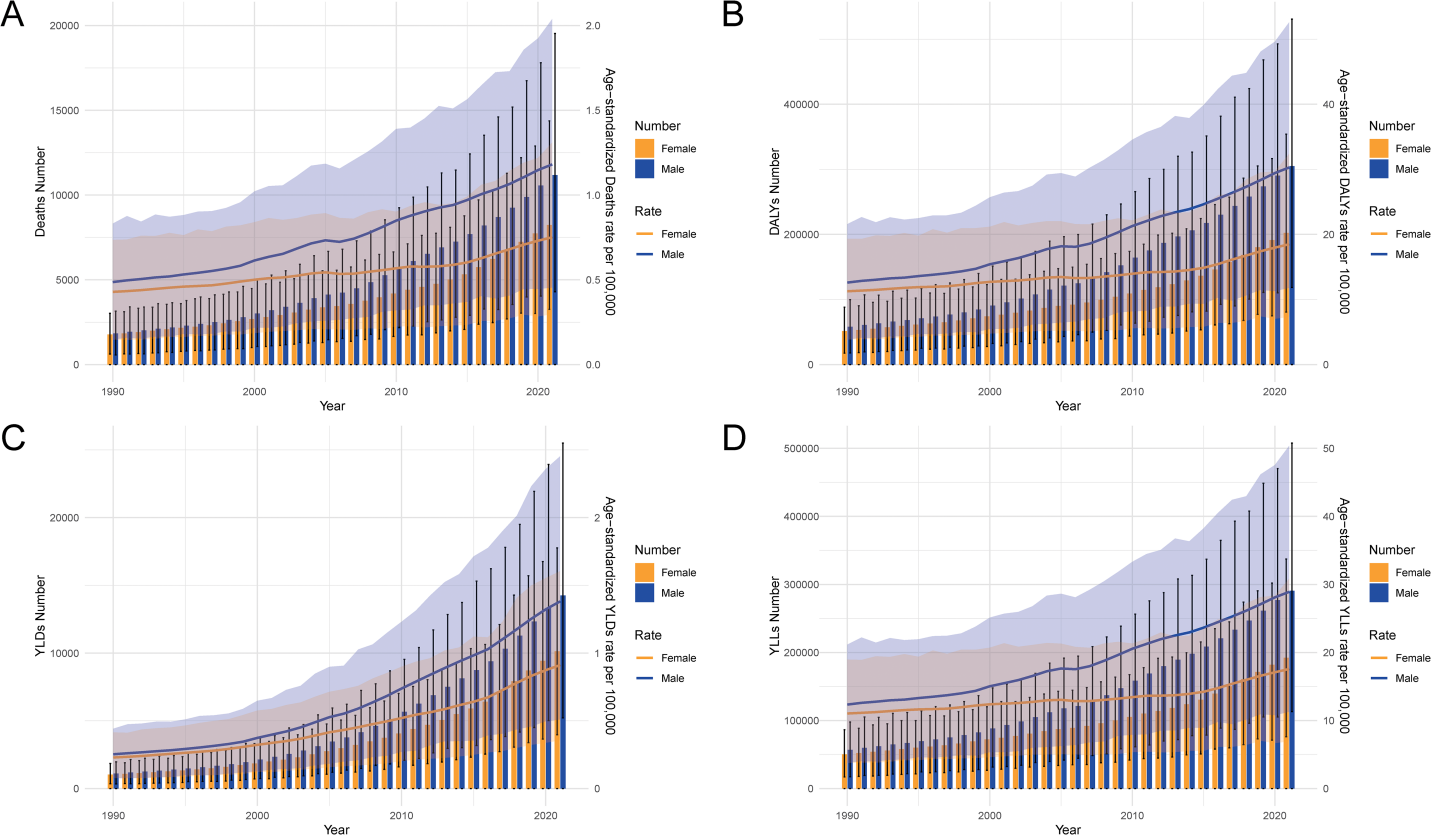
Temporal trends in the numbers and age-standardized rates of deaths, DALYs, YLDs, and YLLs for CRC attributable to high BMI in China from 1990 to 2021. **(A)** The trends in the number of deaths and age-standardized death rates per 100,000 people. **(B)** The trends in DALYs. **(C)** The trends in YLDs. **(D)** The trends in YLLs. DALYs, disability-adjusted life years; YLDs, years lived with disability; YLLs, years of life lost; CRC, colorectal cancer; BMI, body mass index.

The number of deaths increased steadily over time, with males consistently showing higher numbers and age-standardized death rates than females (**Figure 3**). A similar trend is observed in DALYs, where both the number and age-standardized rates have risen significantly, particularly after 2000, with a more pronounced increase in males (**Figure 3**). The non-fatal burden, represented by YLDs, also shows a gradual increase over time for both sexes, although the rise is less steep compared to deaths and DALYs (**Figure 3**). The YLLs, which reflect premature mortality, exhibit the sharpest increase, particularly among males, indicating a growing loss of life years due to CRC attributable to high BMI over the three decades (**Figure 3**).

### Age-specific changes in CRC burden attributable to high BMI between 1990 and 2021

Across all metrics, the burden has significantly increased over time, particularly in older age groups. The number of deaths and crude death rates show a steep rise from age 50 onwards, with a greater increase observed in 2021 compared to 1990 (**Supplementary Figure 1A**). Similarly, DALYs reveal a substantial growth in both the number and crude rate, particularly after age 50, indicating an increasing health burden over the three decades (**Supplementary Figure 1B**). YLDs have risen steadily, reflecting the growing non-fatal burden, especially in middle and older age groups (**Supplementary Figure 1C**). YLLs exhibit the most dramatic increase in older populations, with both the number of years lost and the crude rates rising sharply between 1990 and 2021, particularly after age 60, underscoring the growing impact of high BMI on premature mortality (**Supplementary Figure 1D**).

### Comparison of trends in CRC burden attributable to high BMI between China and global levels

In China, there was a significant increase in all metrics, with the age-standardized death rate rising from 0.45 in 1990 to 1.18 in 2021, a change of 2.43-fold (**Table 2**). Similarly, the DALYs rate increased by 2.33-fold, and YLLs saw a 2.24-fold rise during the same period. The most dramatic increase was observed in YLDs, with a 5.17-fold rise. In contrast, global rates showed more modest increases, with the death rate rising slightly from 1.14 in 1990 to 1.17 in 2021 and a less pronounced rise in DALYs and YLLs compared to China. The global increase in YLDs, although notable at 1.18-fold, was still much smaller than the rise observed in China (**Table 2**, **Supplementary Figure 2**).

**Table 2:** Change of age-standardized rates in deaths, DALYs, YLDs, and YLLs for CRC attributable to high BMI between 1990 and 2021 in China and global level.

Measure	China			Global		
	1990	2021	Change	1990	2021	Change
Deaths	0.45 (0.15, 0.77)	1.18 (0.45, 2.04)	2.43 (2.29 - 2.57) ^*^	1.14 (0.48, 1.86)	1.17 (0.51, 1.87)	0.11 (-0.02 - 0.24)
DALYs	11.88 (4.11, 20.31)	30.29 (11.79, 52.57)	2.33 (2.21 - 2.45) ^*^	25.54 (10.83, 41.2)	27.33 (11.8, 43.37)	0.23 (0.09 - 0.37) ^*^
YLDs	0.24 (0.08, 0.42)	1.38 (0.51, 2.45)	5.17 (4.98 - 5.37) ^*^	0.88 (0.37, 1.48)	1.26 (0.53, 2.09)	1.18 (1.09 - 1.26) ^*^
YLLs	11.64 (4.03, 19.89)	28.9 (11.28, 50.34)	2.24 (2.12 - 2.36) ^*^	24.66 (10.46, 39.77)	26.07 (11.29, 41.49)	0.19 (0.05 - 0.33) ^*^

Values in parentheses indicate 95% uncertainty intervals (UIs), estimated using Monte Carlo simulations. Abbreviations: DALYs, disability-adjusted life-years; YLDs, years lived with disability; YLLs, years of life lost; CRC, colorectal cancer; BMI, body mass index; ^*^, *P*<0.05.

### Trends in age-standardized rates of CRC burden attributable to high BMI in China (1990-2021)

**Figure 4:**
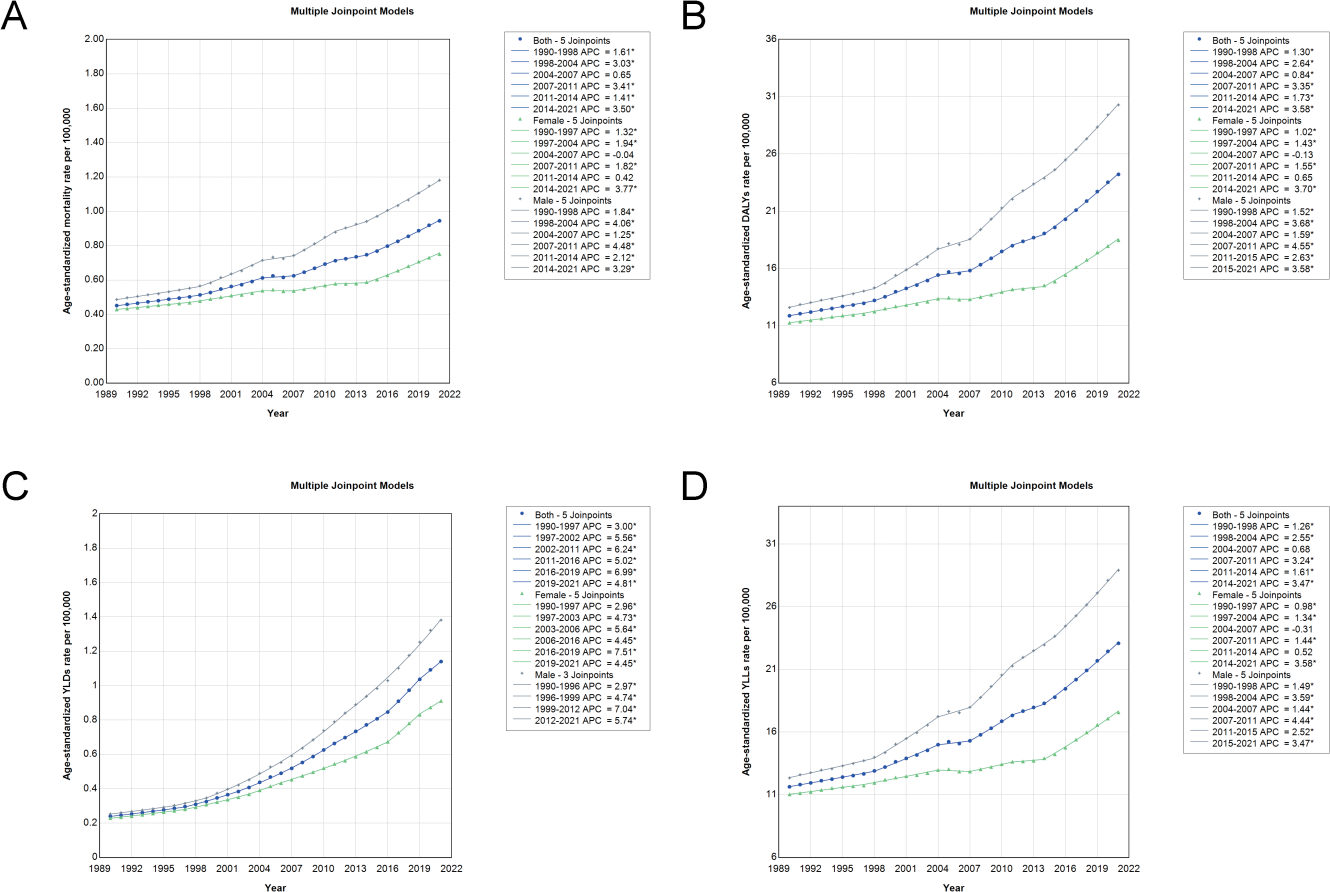
Joinpoint analysis of trends in age-standardized rates of deaths, DALYs, YLDs, and YLLs for CRC attributable to high BMI in China from 1990 to 2021. **(A)** The joinpoint analysis for deaths. **(B)** The analysis for DALYs. **(C)** The analysis for YLDs. **(D)** The analysis for YLLs. DALYs, disability-adjusted life years; YLDs, years lived with disability; YLLs, years of life lost; CRC, colorectal cancer; BMI, body mass index.

Joinpoint regression analysis revealed significant inflection points in the trends of CRC burden attributable to high BMI in China. The ASMR showed an overall increasing trend, with a significant acceleration from 2014 to 2021 (APC: 3.50). A similar upward shift was observed for DALYs during the same period (APC: 3.58) and for YLLs (APC: 3.47), indicating a worsening burden. The most pronounced increase was seen in YLDs between 2016 and 2019 (APC: 6.99), reflecting a growing non-fatal disease burden. Sex-specific trends highlighted that male experienced higher APCs than females across all burden metrics. Notably, for males, the period 2011-2014 exhibited a sharp increase in ASMR (APC: 4.48) and DALYs (APC: 4.55), while the increase among females during the same period was more gradual. Additionally, from 2014 to 2021, the ASMR for females increased significantly (APC: 3.77), demonstrating a widening sex disparity in CRC burden (**Figure 4** and **Supplementary Table 2**).

### Age-period-cohort analysis of CRC burden attributable to high BMI in China

**Figure 5:**
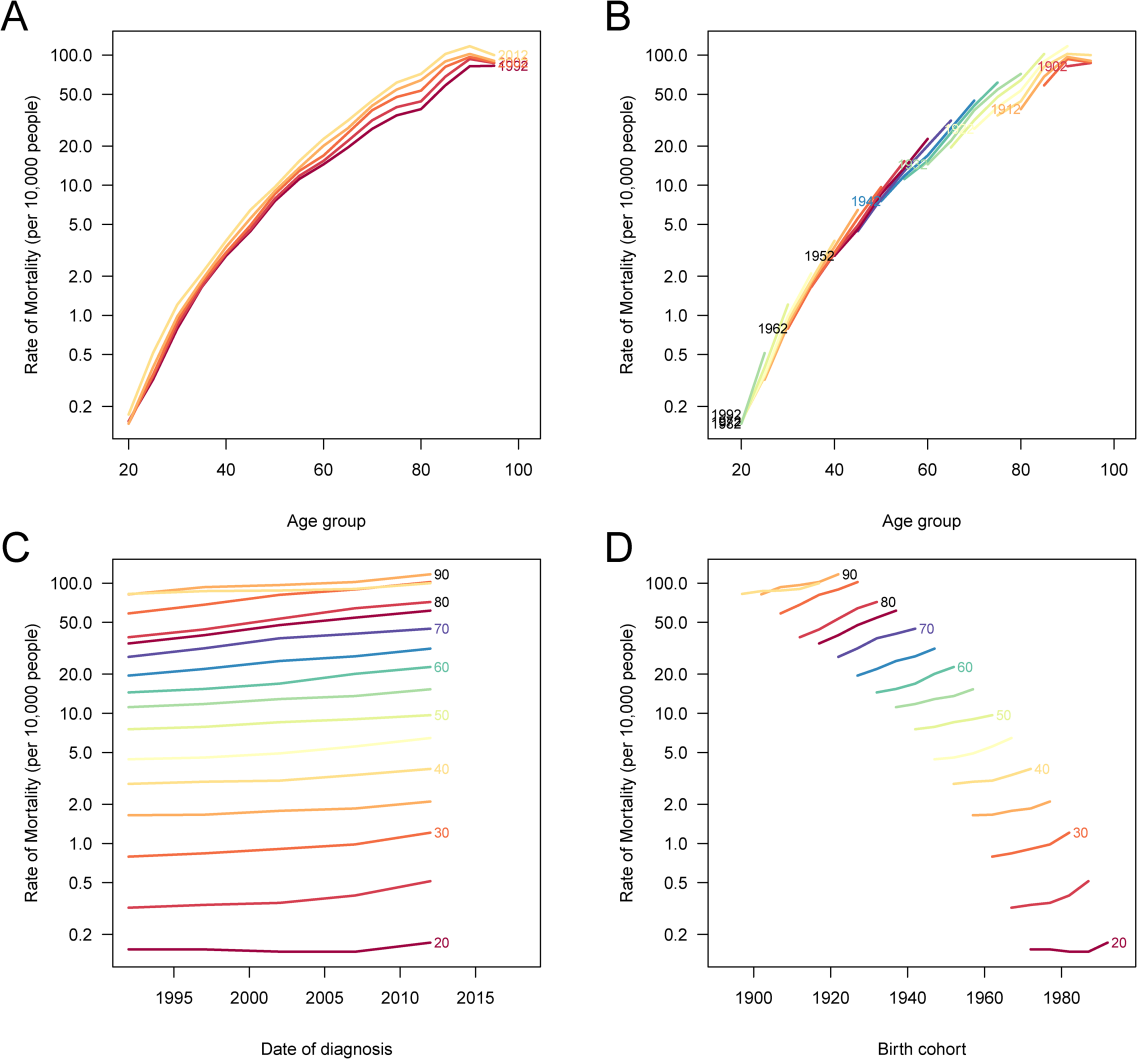
Age-period-cohort (APC) analysis of DALYs for CRC attributable to high BMI in China. **(A)** The age-specific DALY rates of CRC across different time periods; each line connects the age-specific DALY rates for a 5-year period, showing an increasing trend with age. **(B)** The cohort-specific DALY rates of CRC according to birth cohorts; each line connects the cohort-specific DALY rates for a 5-year cohort, with higher risks in more recent cohorts. **(C)** The period-specific DALY rates of CRC across different age groups; each line connects the period-specific DALY rates for a 5-year age group, showing rising DALY rates over time. **(D)** The birth cohort-specific DALY rates of CRC across age groups; each line connects the birth cohort-specific DALY rates for a 5-year age group, illustrating cohort-based variations in DALY rates. DALYs, disability-adjusted life years; CRC, colorectal cancer; BMI, body mass index.

The APC analysis was conducted to assess the independent effects of age, period, and birth cohort on the burden of CRC attributable to high BMI in China. The age effect demonstrates a progressive increase in mortality and DALY rates with advancing age, with the highest burden observed in the elderly population (**Figures 5** and **Supplementary Figure 3A**). This pattern suggests that older individuals face a significantly higher risk of CRC mortality and disability due to the cumulative impact of prolonged exposure to risk factors and age-related physiological changes. The period effect indicates a consistent increase in the relative risk of CRC burden over successive time periods (**Figures 5** and **Supplementary Figure 3C**). This trend reflects advancements in cancer detection and reporting, as well as a growing prevalence of obesity-related metabolic disorders over time. The cohort effect reveals that more recent birth cohorts experience a higher risk of CRC deaths and DALYs attributable to high BMI compared to earlier cohorts (**Figures 5** and **Supplementary Figure 3B**). This finding suggests that individuals born in later decades are exposed to obesogenic environments and sedentary lifestyles from an earlier age, leading to an increased lifetime risk of CRC. Additionally, the cohort-specific age trends further illustrate variations in risk across birth cohorts, showing that younger generations exhibit an elevated risk of CRC as they age (**Figures 5** and **Supplementary Figure 3D**).

### Decomposition analysis of factors contributing to changes in CRC deaths and DALYs attributable to high BMI

**Figure 6:**
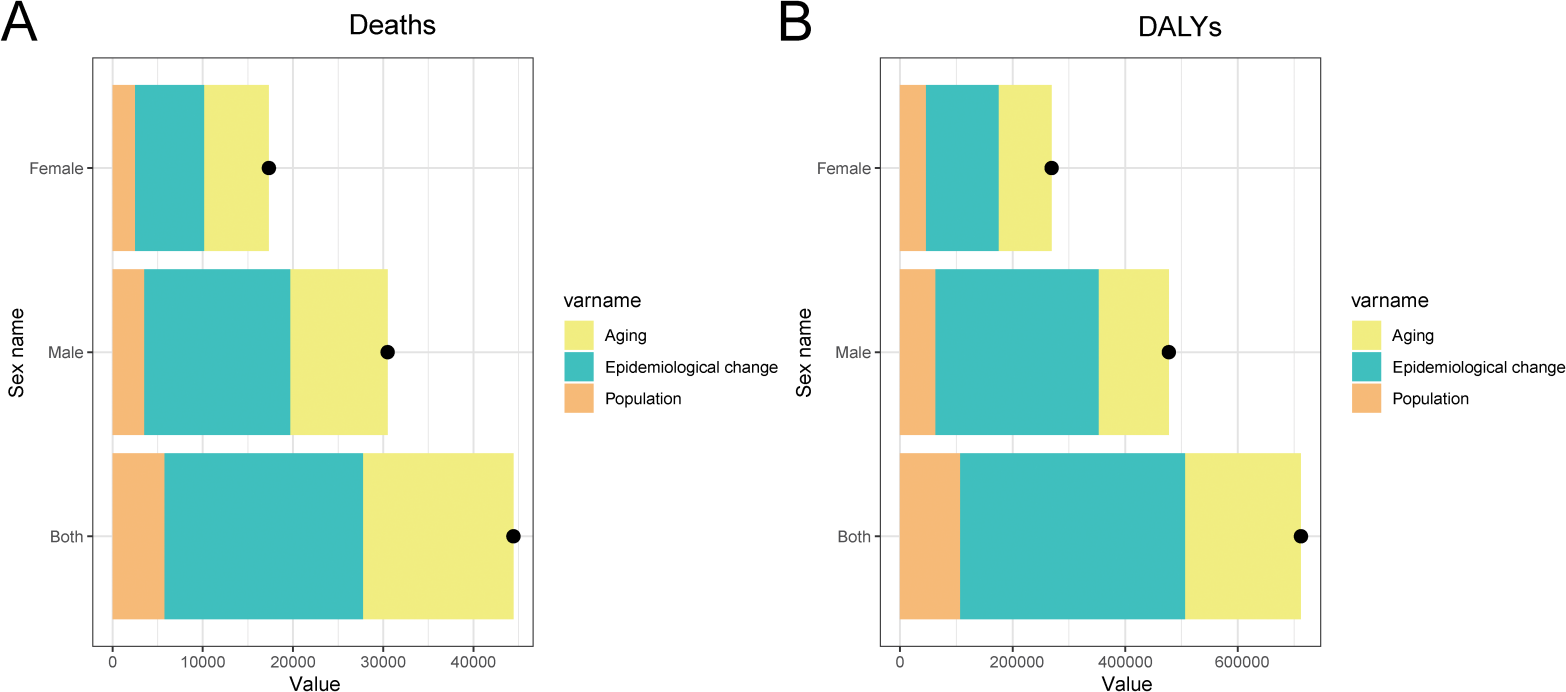
Decomposition analysis of the factors contributing to changes in deaths **(A)** and DALYs **(B)** for CRC attributable to high BMI in China. The contributions are color-coded: population growth (orange), aging (yellow), and epidemiological change (green). The black dots represent the sum of the effects of these three factors.

Epidemiological change had the largest influence on both deaths and DALYs across sexes, followed by aging, while population growth contributed the least. The black dots represent the combined effect of these three factors, illustrating the overall changes in CRC burden. Although epidemiological changes drove the majority of the increase in deaths and DALYs, aging also played a significant role, particularly in males. Population growth had the smallest impact in all groups, but still contributed to the overall increase in CRC burden (**Figure 6**).

## Discussion

### Major findings

This study provides a comprehensive assessment of the burden of CRC attributable to high BMI in China from 1990 to 2021. The findings indicate a substantial increase in CRC-related deaths and DALYs over the past three decades, with a 2.43-fold rise in ASMR and a 2.33-fold increase in DALYs. Males consistently experienced a higher burden compared to females, particularly in terms of YLLs and DALYs. Decomposition analysis identified epidemiological changes as the primary driver behind the increasing CRC burden, followed by aging, while population growth played a minor role. Furthermore, APC analysis highlighted that more recent birth cohorts faced higher risks of CRC attributable to high BMI, particularly in older age groups. These findings underscore the urgent need for targeted interventions to mitigate the growing impact of obesity on CRC burden in China.

### Socioeconomic drivers and lifestyle transitions

The findings of this study are consistent with global trends but underscore a more rapid increase in CRC burden in China compared to other countries. Prior studies estimate that high BMI accounts for 8-13% of all CRC cases globally (22), with recent data indicating a growing CRC burden attributable to obesity, particularly in nations undergoing rapid economic and lifestyle transitions, including China (23). The steeper increase in CRC burden in China compared to global averages reflects an accelerated rise in obesity rates, driven by urbanization, dietary shifts, and sedentary lifestyles. Unlike many high-income countries where obesity rates have plateaued, China continues to experience a sharp increase in obesity prevalence, exacerbating the burden of CRC. This study expands upon previous research by integrating long-term trend analysis, sex-specific comparisons, and decomposition analysis, offering a comprehensive epidemiological perspective on the role of high BMI in CRC development.

The substantial increase in CRC burden attributable to high BMI in China is largely fueled by shifting lifestyle and behavioral patterns. Rapid urbanization has led to a Westernization of dietary habits, with increased consumption of energy-dense, processed foods high in saturated fats, refined sugars, and sodium, alongside a decline in fiber-rich foods such as vegetables, fruits, and whole grains (24–26). These dietary shifts contribute to obesity and metabolic dysfunction, both of which elevate CRC risk. Additionally, the food environment in China has changed drastically over the past three decades, with greater availability of ultra-processed foods, aggressive marketing of unhealthy products, and a decline in traditional home-cooked meals. Combined with an increasingly sedentary lifestyle due to widespread adoption of technology and reductions in physically active occupations, these factors have created an obesogenic environment that significantly impacts CRC burden. Addressing these trends requires comprehensive public health interventions that promote healthier dietary patterns and increased physical activity.

### Rising YLDs and improved disease surveillance

The significant increase in YLDs (5.17-fold) in this study highlights the growing non-fatal burden of CRC, which may be attributed to several factors. Advances in cancer detection and treatment have improved survival rates, allowing more individuals to live with CRC and its long-term complications. Increased disease awareness and enhanced surveillance systems have likely contributed to the higher identification of CRC cases, particularly at earlier stages, which, while improving prognosis, also results in prolonged disease management and disability (27). Additionally, obesity itself is associated with metabolic dysfunction, chronic inflammation, and a higher likelihood of treatment-related complications, all of which can contribute to a greater non-fatal burden among affected individuals (28).

### Biological mechanisms linking high BMI to CRC

Sex-specific differences observed in this study, where males exhibited a higher CRC burden, may be explained by biological and behavioral factors. Males tend to accumulate visceral adiposity, which is metabolically more active than subcutaneous fat and promotes a chronic pro-inflammatory state. Increased levels of tumor necrosis factor-alpha (TNF-α), interleukin-6 (IL-6), and C-reactive protein (CRP) contribute to insulin resistance, oxidative stress, and enhanced cellular proliferation, creating a microenvironment conducive to CRC development and progression. Additionally, estrogen is known to exert protective effects in females by modulating immune responses, reducing inflammation, and improving insulin sensitivity, potentially lowering CRC risk in premenopausal women (29, 30). Behavioral differences, such as lower participation in preventive health measures and CRC screening among males, may further contribute to the observed disparities in disease burden. These findings align with previous studies emphasizing sex-based disparities in CRC incidence and outcomes (22, 31).

Furthermore, the APC analysis suggests that cohort effects significantly contribute to CRC burden trends. Younger generations with early-life exposure to obesity-related risk factors face a higher cumulative CRC risk over time (32, 33). This underscores the need for early intervention strategies targeting childhood and adolescent obesity to mitigate the long-term impact of high BMI on CRC incidence. Without effective public health measures, the increasing prevalence of obesity among younger populations may drive a sustained rise in CRC burden, reinforcing the urgency of implementing nationwide preventive strategies. High BMI contributes to colorectal carcinogenesis through multiple biological pathways, including chronic low-grade inflammation, insulin resistance, and gut microbiome alterations (34, 35). Adipose tissue secretes pro-inflammatory cytokines, such as TNF-α and interleukins, creating a pro-carcinogenic environment (36, 37). Elevated insulin and insulin-like growth factor (IGF) levels in individuals with obesity further promote cellular proliferation and inhibit apoptosis, increasing tumorigenesis risk (38). Additionally, obesity-induced gut microbiome dysbiosis may contribute to intestinal inflammation and CRC progression (39, 40). These mechanisms highlight the need for primary prevention strategies, including weight management, healthy dietary interventions, and regular physical activity to reduce CRC risk in high-risk populations.

### Policy implications

To effectively reduce the CRC burden attributable to high BMI, urgent, multi-sectoral public health actions and policy interventions are necessary. Comprehensive obesity prevention strategies should focus on nationwide nutrition policies, including front-of-pack labeling, trans-fat bans, and taxation on sugar-sweetened beverages, which have been effective in reducing the consumption of unhealthy processed foods. Expanding BMI screening programs and implementing targeted public health campaigns promoting fiber-rich, plant-based diets while discouraging excessive intake of processed and high-fat foods are crucial components. Encouraging physical activity through urban planning initiatives, such as increasing access to green spaces, developing walkable environments, and enhancing active transportation infrastructure, can help counteract the sedentary lifestyle associated with obesity. Additionally, workplace wellness programs, community-based exercise initiatives, and school-based health education should be reinforced to establish long-term behavioral changes (41). Routine CRC screening programs should also be enhanced through risk-stratified approaches, particularly for individuals with high BMI and metabolic comorbidities, as early detection through fecal immunochemical testing (FIT) and colonoscopy significantly reduces CRC mortality. Integrating personalized CRC risk assessments into routine healthcare and ensuring equitable access to screening services can improve early diagnosis and outcomes. Furthermore, policy measures targeting the social determinants of health, such as ensuring equitable access to affordable, nutritious foods and safe exercise facilities, are essential to reducing health disparities and mitigating the growing burden of CRC in China (42).

### Limitations and need for subnational analysis

Despite the strengths of this study, several limitations must be acknowledged. The reliance on GBD-modeled estimates rather than individual patient-level data may introduce uncertainties related to data quality and regional variations, particularly in earlier years when registry coverage was more limited. The study did not account for other CRC risk factors, such as smoking, alcohol consumption, dietary composition beyond BMI, and genetic predisposition, which could provide a more comprehensive risk assessment. Additionally, the population-level analysis rather than individual-level cohort studies limits the ability to examine personalized risk profiles and gene-environment interactions. Furthermore, the study did not evaluate the effectiveness of existing public health policies aimed at reducing obesity rates and CRC burden. Future research should focus on prospective cohort studies examining the long-term effects of BMI on CRC risk, as well as randomized controlled trials (RCTs) assessing the impact of weight-loss interventions, dietary modifications, and structured exercise programs in CRC prevention. Additionally, longitudinal studies evaluating the real-world impact of national obesity prevention policies - such as taxation policies, front-of-pack labeling, and public awareness campaigns - are needed to determine their effectiveness in reducing CRC incidence. A more granular, subnational analysis incorporating regional disparities in obesity trends, dietary habits, and healthcare access will further strengthen the evidence base for policy-driven interventions.

## Conclusions

This study underscores the significant public health challenge posed by the rising burden of CRC attributable to high BMI in China. Addressing this issue requires multifaceted public health interventions focused on obesity prevention and control. Moving forward, more research is needed to explore the combined effects of high BMI and other modifiable risk factors, such as diet and physical activity, on CRC risk. Future studies should also examine the long-term effectiveness of public health policies aimed at reducing obesity and CRC burden. In addition, the role of personalized interventions based on genetic, lifestyle, and socioeconomic factors should be considered to tailor prevention strategies more effectively. As the population continues to age, the ongoing monitoring of CRC trends will be critical for informing future policy decisions and healthcare resource allocation to mitigate the burden of obesity-related cancers.

## Data Availability

Publicly available datasets were analyzed in this study. This data can be found here: http://ghdx.healthdata.org/gbd-results-tool.

## Supplementary material

Supplementary Figure 1 Comparison of the numbers and crude rates of deaths, DALYs, YLDs, and YLLs for CRC attributable to high BMI by age group in China, 1990 and 2021. **(A)** The number of deaths and crude death rates per 100,000 people. **(B)** The number of DALYs and crude DALY rates per 100,000 people. **(C)** The number of YLDs and crude YLD rates per 100,000 people. **(D)** The number of YLLs and crude YLL rates per 100,000 people. DALYs, disability-adjusted life years; YLDs, years lived with disability; YLLs, years of life lost; CRC, colorectal cancer; BMI, body mass index.

Supplementary Figure 2 Trends in age-standardized rates of deaths, DALYs, YLDs, and YLLs for CRC attributable to high BMI in China and globally from 1990 to 2021. **(A)** Global age-standardized rates per 100,000 people from 1990 to 2021. **(B)** China-specific age-standardized rates per 100,000 people during the same period. DALYs, disability-adjusted life years; YLDs, years lived with disability; YLLs, years of life lost; CRC, colorectal cancer; BMI, body mass index.

Supplementary Figure 3 Age-period-cohort (APC) analysis of deaths for CRC attributable to high BMI in China. **(A)** The age-specific mortality rates of CRC across different time periods; each line connects the age-specific mortality rates for a 5-year period, showing an increasing trend with age. **(B)** The cohort-specific mortality rates of CRC according to birth cohorts; each line connects the cohort-specific mortality rates for a 5-year cohort, indicating a higher risk in more recent cohorts. **(C)** The period-specific mortality rates of CRC across different age groups; each line connects the period-specific mortality rates for a 5-year age group. **(D)** The birth cohort-specific mortality rates of CRC across age groups; each line connects the birth cohort-specific mortality rates for a 5-year age group, revealing cohort-based variations in mortality risk. CRC, colorectal cancer; BMI, body mass index.

## References

[1] Morgan E Arnold M Gini A Lorenzoni V Cabasag CJ Laversanne M. Global burden of colorectal cancer in 2020 and 2040: incidence and mortality estimates from GLOBOCAN. Gut. (2023) 72:338–44. doi: 10.1136/gutjnl-2022-327736 36604116

[2] Zhan Z Chen B Lin W Chen X Huang R Yang C. Rising burden of colon and rectum cancer in China: an analysis of trends, gender disparities, and projections to 2030. Ann Surg Oncol. (2025) 32:3361–71. doi: 10.1245/s10434-025-16905-w 39836276

[3] Zhan Z Chen B Lin W Chen X Huang R Yang C. ASO author reflections: addressing the rising burden of colon and rectum cancer in China: gender disparities, trends, and projections. Ann Surg Oncol. (2025) 32:3408–9. doi: 10.1245/s10434-025-16948-z 39873849

[4] Cairns BJ. Cancer and high body-mass index: global burden, global effort? Lancet Oncol. (2015) 16:3–4. doi: 10.1016/S1470-2045(14)70373-0 25467405

[5] Chen X Li H Mandic M Hoffmeister M Brenner H. Assessment of body mass index, polygenic risk score, and development of colorectal cancer. JAMA network Open. (2022) 5:e2248447. doi: 10.1001/jamanetworkopen.2022.48447 36547977 PMC9857417

[6] Blüher M. Obesity: global epidemiology and pathogenesis. Nat Rev Endocrinol. (2019) 15:288–98. doi: 10.1038/s41574-019-0176-8 30814686

[7] Sung H Siegel RL Torre LA Pearson-Stuttard J Islami F Fedewa SA. Jacobs EJ et al: Global patterns in excess body weight and the associated cancer burden. CA: A Cancer J clinicians. (2019) 69:88–112. doi: 10.3322/caac.21499 30548482

[8] Aleksandrova K Pischon T Buijsse B May AM Peeters PH Bueno-de-Mesquita HB. Siersema PD et al: Adult weight change and risk of colorectal cancer in the European Prospective Investigation into Cancer and Nutrition. Eur J Cancer (Oxford England: 1990). (2013) 49:3526–36. doi: 10.1016/j.ejca.2013.06.021 23867126

[9] Carr PR Amitay EL Jansen L Alwers E Roth W Herpel E. Chang-Claude J et al: Association of BMI and major molecular pathological markers of colorectal cancer in men and women. Am J Clin Nutr. (2020) 111:562–9. doi: 10.1093/ajcn/nqz315 31897467

[10] Safizadeh F Mandic M Pulte D Niedermaier T Hoffmeister M Brenner H. The underestimated impact of excess body weight on colorectal cancer risk: Evidence from the UK Biobank cohort. Br J Cancer. (2023) 129:829–37. doi: 10.1038/s41416-023-02351-6 PMC10449928 37443347

[11] Saeed U Myklebust T Robsahm TE Kielland MF Møller B Skålhegg BS. Risk and survival in colorectal cancer with increasing body mass index: A nationwide population-based cohort study. Colorectal Dis. (2023) 25:375–85. doi: 10.1111/codi.16367 36222384

[12] Huang L Wang Z Wang H Zhao L Jiang H Zhang B. Nutrition transition and related health challenges over decades in China. Eur J Clin Nutr. (2021) 75:247–52. doi: 10.1038/s41430-020-0674-8 32620907

[13] GBD 2021 Diseases and Injuries Collaborators. Global incidence, prevalence, years lived with disability (YLDs), disability-adjusted life-years (DALYs), and healthy life expectancy (HALE) for 371 diseases and injuries in 204 countries and territories and 811 subnational locations, 1990-2021: a systematic analysis for the Global Burden of Disease Study 2021. Lancet (London England). (2024) 403:2133–61. doi: 10.1016/S0140-6736(24)00757-8 PMC11122111 38642570

[14] Mao Q Tian X Wang X Xu H Zhang Y Kong Y. Global burden of cardiovascular diseases attributable to diet low in seafood omega-3 fatty acids from 1990~2021 and forecasting the future trends: A population-based study. PLoS One. (2025) 20:e0316767. doi: 10.1371/journal.pone.0316767 39908338 PMC12051498

[15] Zhan Z Chen X Zheng J Xu J Zhou S Guo Z. Burden of colon and rectum cancer attributable to processed meat consumption in China, 1990-2021. Frontiers in Nutrition. (2025) 12:1488077. doi: 10.3389/fnut.2025.1488077 40225336 PMC11985440

[16] Dai H Alsalhe TA Chalghaf N Riccò M Bragazzi NL Wu J. The global burden of disease attributable to high body mass index in 195 countries and territories, 1990-2017: An analysis of the Global Burden of Disease Study. PLoS Med. (2020) 17:e1003198. doi: 10.1371/journal.pmed.1003198 32722671 PMC7386577

[17] GBD 2019 Colorectal Cancer Collaborators. Global, regional, and national burden of colorectal cancer and its risk factors, 1990-2019: a systematic analysis for the Global Burden of Disease Study 2019. Lancet Gastroenterology hepatology. (2022) 7:627–47. doi: 10.1016/S2468-1253(22)00044-9 PMC9192760 35397795

[18] Kim HJ Fay MP Feuer EJ Midthune DN. Permutation tests for joinpoint regression with applications to cancer rates. Stat Med. (2000) 19:335–51. doi: 10.1002/(SICI)1097-0258(20000215)19:3<335::AID-SIM336>3.0.CO;2-Z 10649300

[19] Kim S Lee S Choi JI Cho H. Binary genetic algorithm for optimal joinpoint detection: Application to cancer trend analysis. Stat Med. (2021) 40:799–822. doi: 10.1002/sim.v40.3 33205511

[20] Li Y Ning Y Shen B Shi Y Song N Fang Y. Temporal trends in prevalence and mortality for chronic kidney disease in China from 1990 to 2019: an analysis of the Global Burden of Disease Study 2019. Clin Kidney J. (2023) 16:312–21. doi: 10.1093/ckj/sfac218 PMC9900593 36755850

[21] Bardo AR Reither EN. Age-period-cohort models. In: Gu D Dupre ME, editors. Encyclopedia of Gerontology and Population Aging. Springer International Publishing, Cham (2020). p. 1–8.

[22] Arnold M Pandeya N Byrnes G Renehan PAG Stevens GA Ezzati PM. Dikshit R et al: Global burden of cancer attributable to high body-mass index in 2012: a population-based study. Lancet Oncol. (2015) 16:36–46. doi: 10.1016/S1470-2045(14)71123-4 25467404 PMC4314462

[23] Bray F Laversanne M Sung H Ferlay J Siegel RL Soerjomataram I. Global cancer statistics 2022: GLOBOCAN estimates of incidence and mortality worldwide for 36 cancers in 185 countries. CA: A Cancer J clinicians. (2024) 74:229–63. doi: 10.3322/caac.21834 38572751

[24] Wang L Zhou B Zhao Z Yang L Zhang M Jiang Y. Huang Z et al: Body-mass index and obesity in urban and rural China: findings from consecutive nationally representative surveys during 2004-18. Lancet (London England). (2021) 398:53–63. doi: 10.1016/S0140-6736(21)00798-4 34217401 PMC7617101

[25] Sun X Yan AF Shi Z Zhao B Yan N Li K. Cheskin LJ et al: Health consequences of obesity and projected future obesity health burden in China. Obesity (Silver Spring Md). (2022) 30:1724–51. doi: 10.1002/oby.23472 36000246

[26] Hemmingsson E. The unparalleled rise of obesity in China: a call to action. Int J Obesity. (2021) 45:921–2. doi: 10.1038/s41366-021-00774-w 33608648

[27] Shinji S Yamada T Matsuda A Sonoda H Ohta R Iwai T. Recent advances in the treatment of colorectal cancer: A review. J Nippon Med School = Nippon Ika Daigaku zasshi. (2022) 89:246–54. doi: 10.1272/jnms.JNMS.2022_89-310 35082204

[28] Zhai W Yang Y Zhang K Sun L Luo M Han X. Impact of visceral obesity on infectious complications after resection for colorectal cancer: a retrospective cohort study. Lipids Health Dis. (2023) 22:139. doi: 10.1186/s12944-023-01890-4 37653410 PMC10469994

[29] Conti L Del Cornò M Gessani S. Revisiting the impact of lifestyle on colorectal cancer risk in a gender perspective. Crit Rev oncology/hematology. (2020) 145:102834. doi: 10.1016/j.critrevonc.2019.102834 31790930

[30] Williams C DiLeo A Niv Y Gustafsson J. Estrogen receptor beta as target for colorectal cancer prevention. Cancer Lett. (2016) 372:48–56. doi: 10.1016/j.canlet.2015.12.009 26708506 PMC4744541

[31] Li Q Wu H Cao M Li H He S Yang F. Xia C et al: Colorectal cancer burden, trends and risk factors in China: A review and comparison with the United States. Chin J Cancer Res = Chung-kuo yen cheng yen chiu. (2022) 34:483–95. doi: 10.21147/j.issn.1000-9604.2022.05.08 PMC9646460 36398126

[32] Printz C. Obesity-related cancers are increasing in young adults. Cancer. (2019) 125:2147–7. doi: 10.1002/cncr.32299 32162683

[33] Liu C Yuan YC Guo MN Xin Z Chen GJ Ding N. Rising incidence of obesity-related cancers among younger adults in China: A population-based analysis (2007-2021). Med (New York NY). (2024) 5(11):1402–1412.e2. doi: 10.1016/j.medj.2024.07.012 PMC11560649 39181132

[34] Riondino S Roselli M Palmirotta R Della-Morte D Ferroni P Guadagni F. Obesity and colorectal cancer: role of adipokines in tumor initiation and progression. World J gastroenterology. (2014) 20:5177–90. doi: 10.3748/wjg.v20.i18.5177 PMC4017033 24833848

[35] Avgerinos KI Spyrou N Mantzoros CS Dalamaga M. Obesity and cancer risk: Emerging biological mechanisms and perspectives. Metabolism. (2019) 92:121–35. doi: 10.1016/j.metabol.2018.11.001 30445141

[36] Sethi JK Hotamisligil GS. Metabolic Messengers: tumour necrosis factor. Nat Metab. (2021) 3:1302–12. doi: 10.1038/s42255-021-00470-z 34650277

[37] Macleod A Scheurlen KM Burton JF Parks MA Sumy MSA Gaskins JT. Systemic adiponectin levels in colorectal cancer and adenoma: a systematic review and meta-analysis. Int J Obesity. (2023) 47:911–21. doi: 10.1038/s41366-023-01358-6 37626126

[38] Hua H Kong Q Yin J Zhang J Jiang Y. Insulin-like growth factor receptor signaling in tumorigenesis and drug resistance: a challenge for cancer therapy. J Hematol Oncol. (2020) 13:64. doi: 10.1186/s13045-020-00904-3 32493414 PMC7268628

[39] Shoji M Sasaki Y Abe Y Nishise S Yaoita T Yagi M. Sakai T et al: Characteristics of the gut microbiome profile in obese patients with colorectal cancer. JGH Open. (2021) 5:498–507. doi: 10.1002/jgh3.12529 33860101 PMC8035457

[40] Sánchez-Alcoholado L Ordóñez R Otero A Plaza-Andrade I Laborda-Illanes A Medina JA. Gut microbiota-mediated inflammation and gut permeability in patients with obesity and colorectal cancer. Int J Mol Sci. (2020) 21:6782. doi: 10.3390/ijms21186782 32947866 PMC7555154

[41] Swinburn BA Kraak VI Allender S Atkins VJ Baker PI Bogard JR. Devarajan R et al: The Global Syndemic of Obesity, Undernutrition, and Climate Change: The Lancet Commission report. Lancet (London England). (2019) 393:791–846. doi: 10.1016/S0140-6736(18)32822-8 30700377

[42] Helsingen LM Vandvik PO Jodal HC Agoritsas T Lytvyn L Anderson JC. Colorectal cancer screening with faecal immunochemical testing, sigmoidoscopy or colonoscopy: a clinical practice guideline. BMJ. (2019) 367:l5515. doi: 10.1136/bmj.l5515 31578196

