# Supplementary figures and images for: Rising colorectal cancer burden attributable to high body mass index in China from 1990 to 2021: a comprehensive analysis using the global burden of disease study
Source: Front Endocrinol (Lausanne). 2025 May 15;16:1509497. doi: 10.3389/fendo.2025.1509497 (PMC12119292; doi:10.3389/fendo.2025.1509497)

A

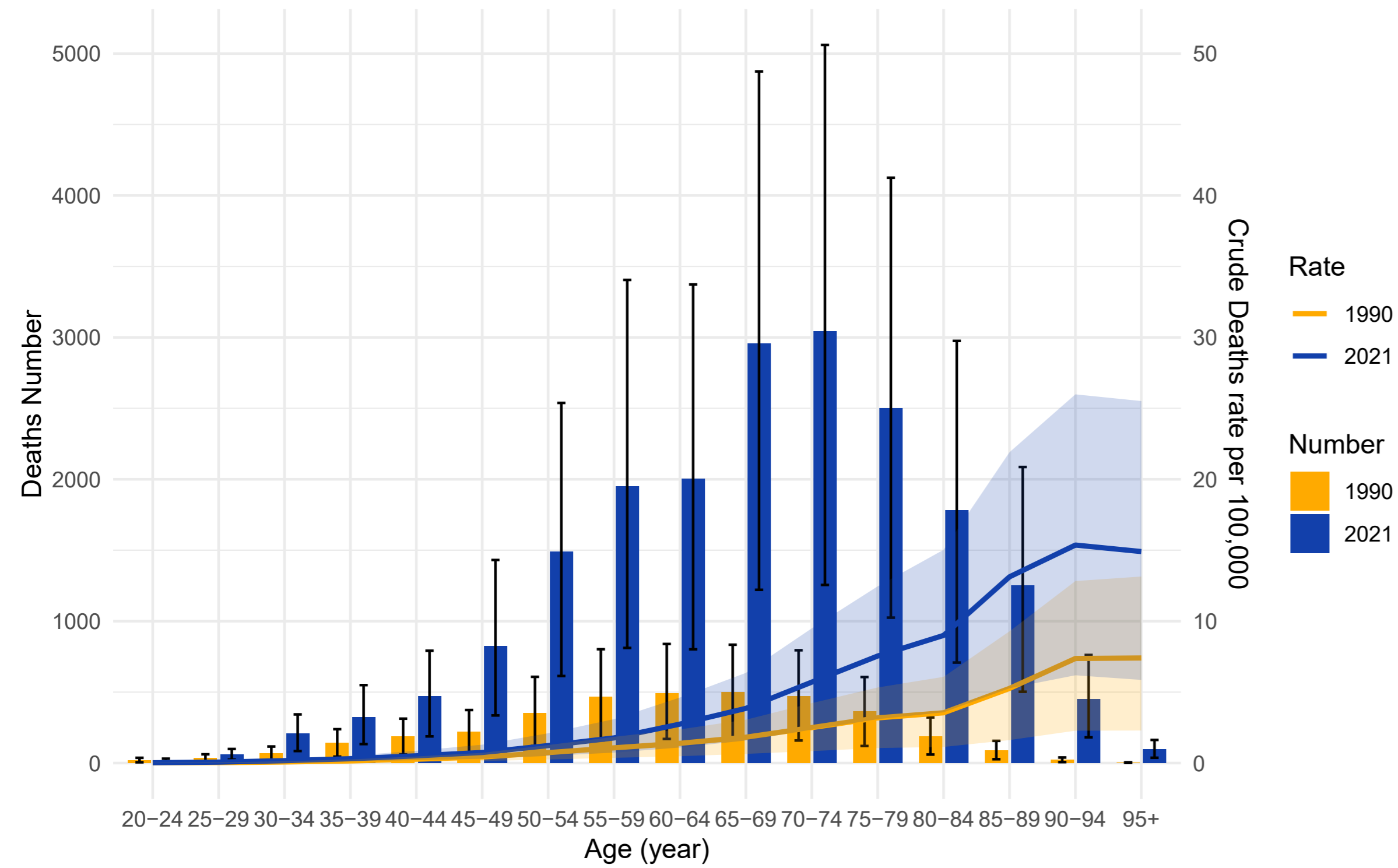

B

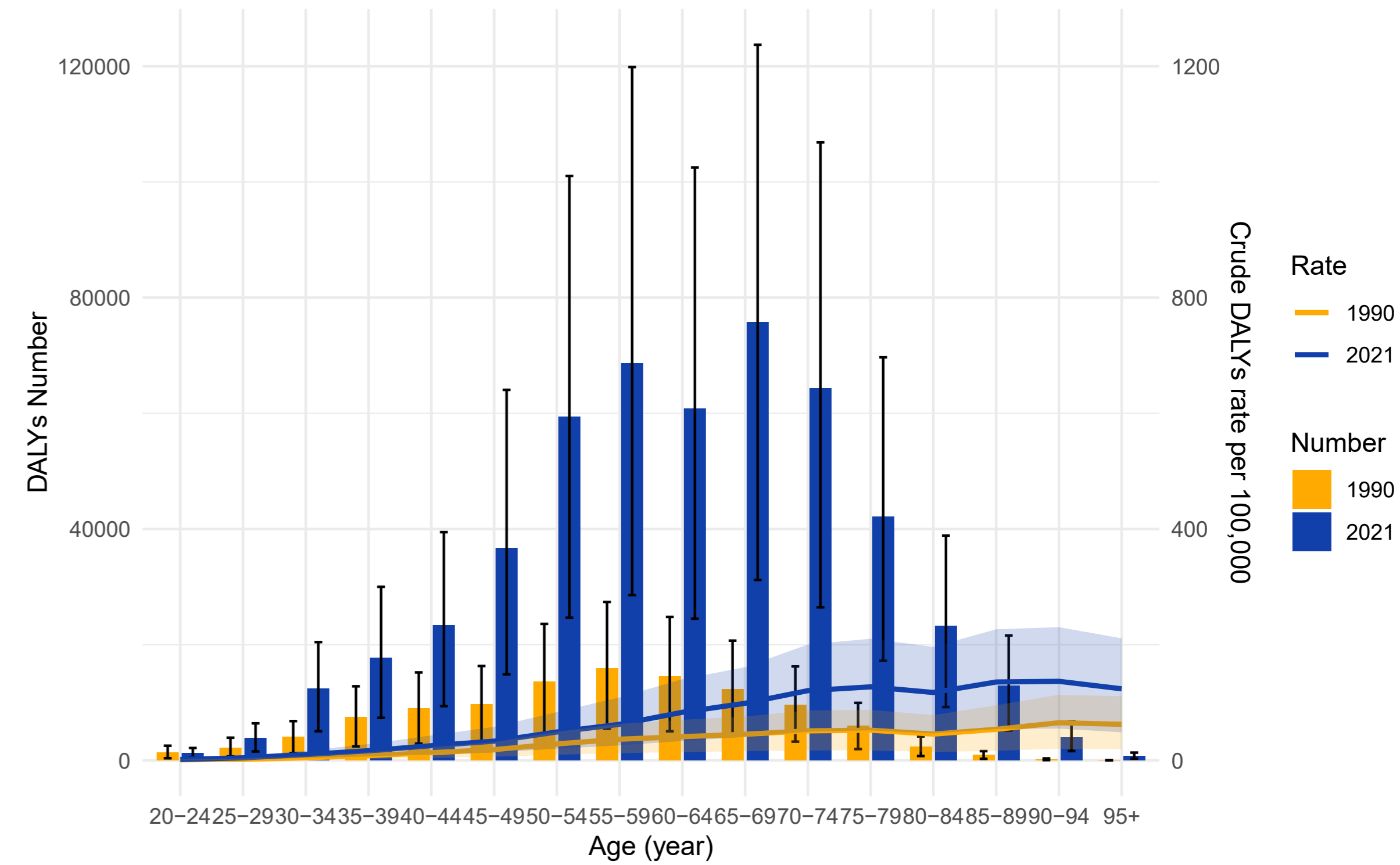

C

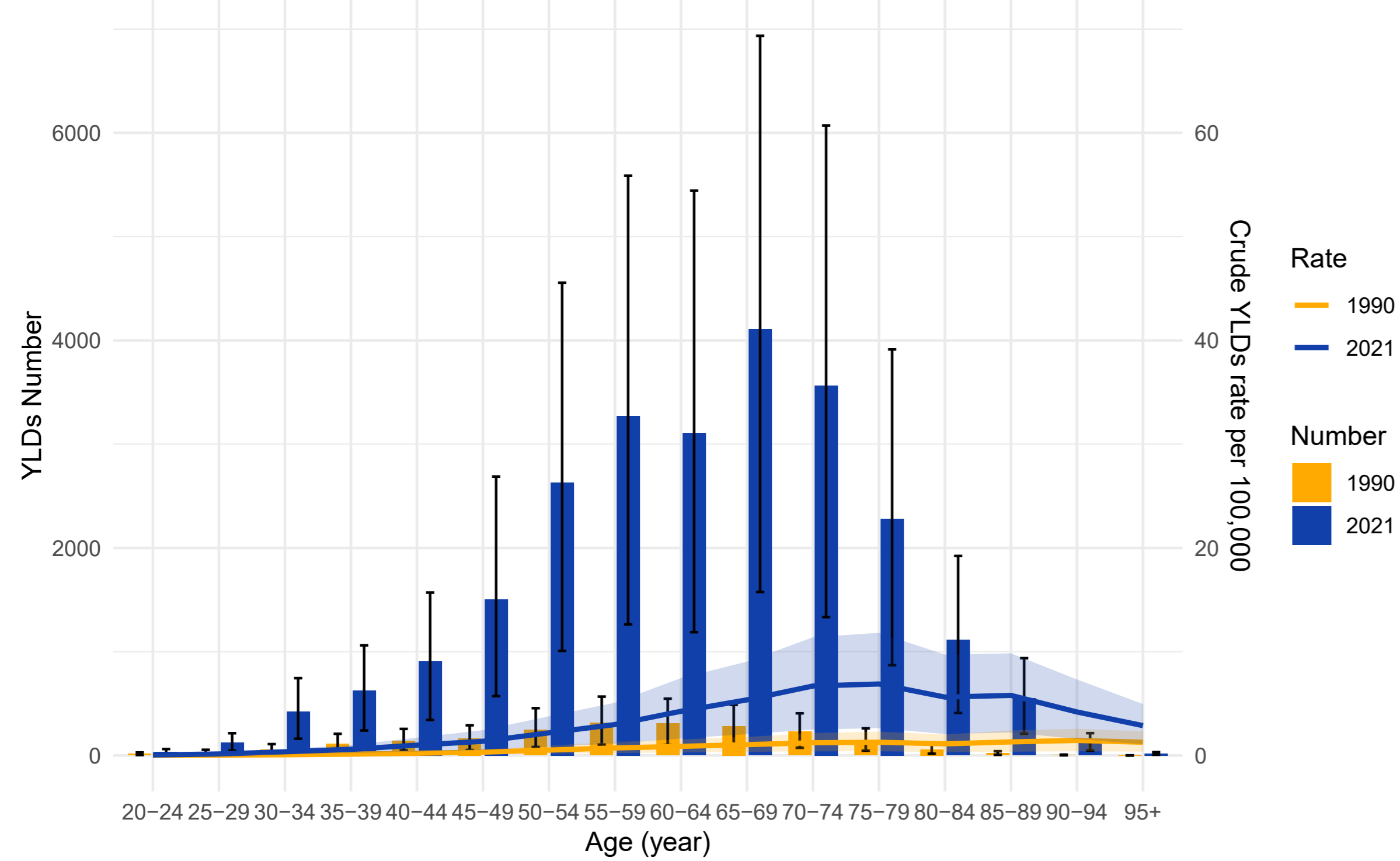

D

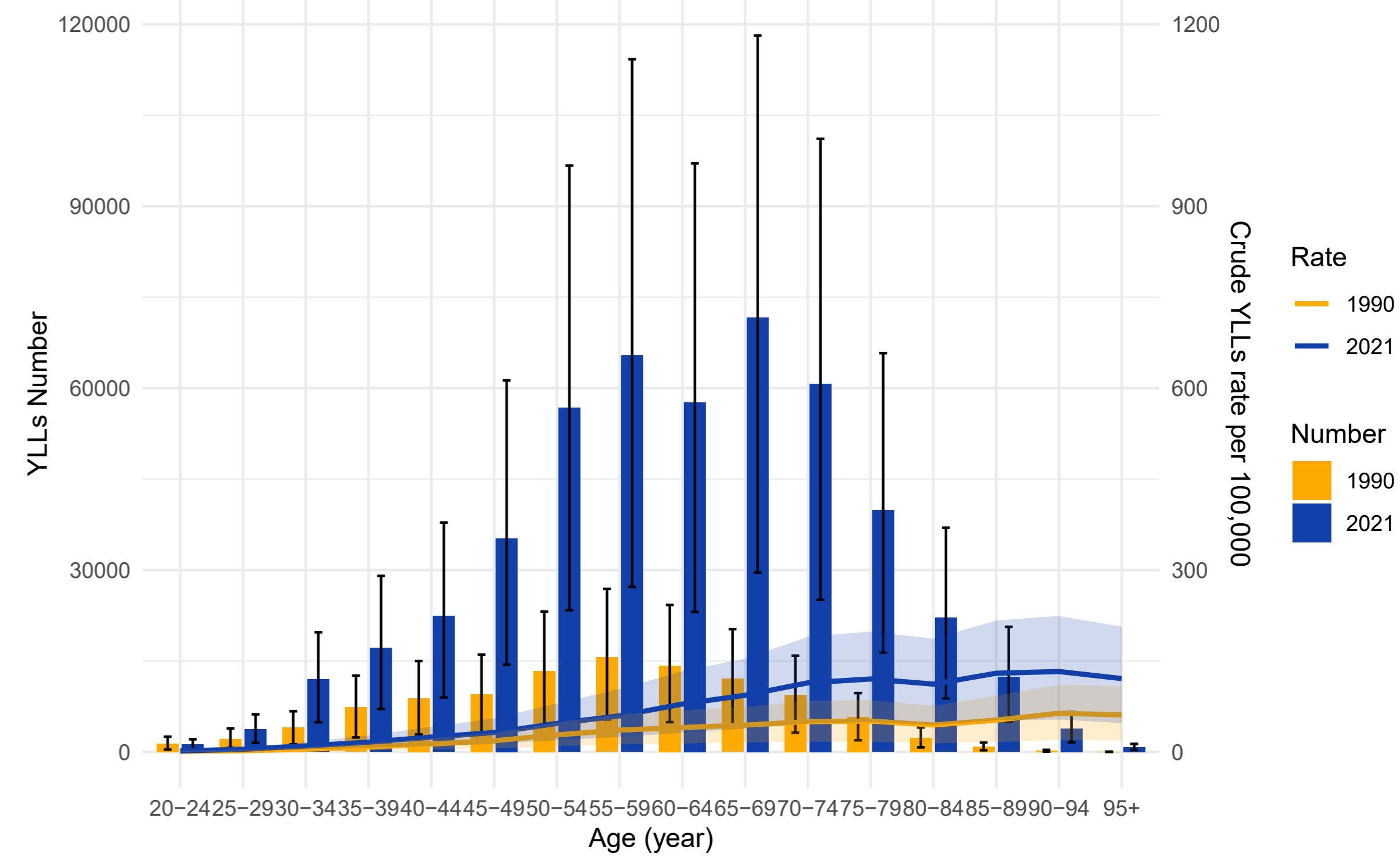

Supplement: Supplementary Figure 1 — Comparison of the numbers and crude rates of deaths, DALYs, YLDs, and YLLs for CRC attributable to high BMI by age group in China, 1990 and 2021. (A) The number of deaths and crude death rates per 100,000 people. (B) The number of DALYs and crude DALY rates per 100,000 people. (C) The number of YLDs and crude YLD rates per 100,000 people. (D) The number of YLLs and crude YLL rates per 100,000 people. DALYs, disability-adjusted life years; YLDs, years lived with disability; YLLs, years of life lost; CRC, colorectal cancer; BMI, body mass index. [file DataSheet1.pdf]

A

1990–2021 Global Age-standardized Rate

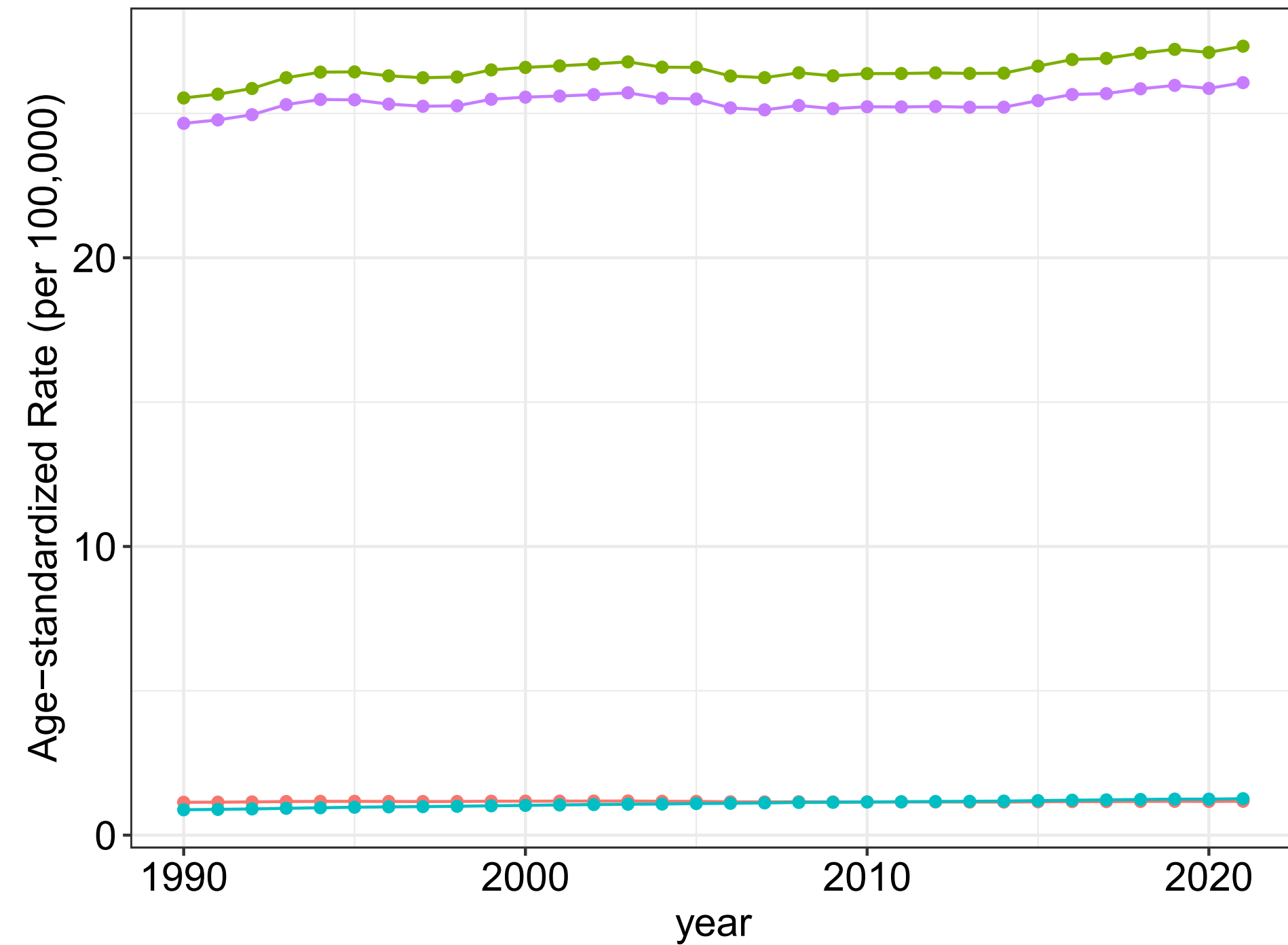

B

1990–2021 China Age-standardized Rate

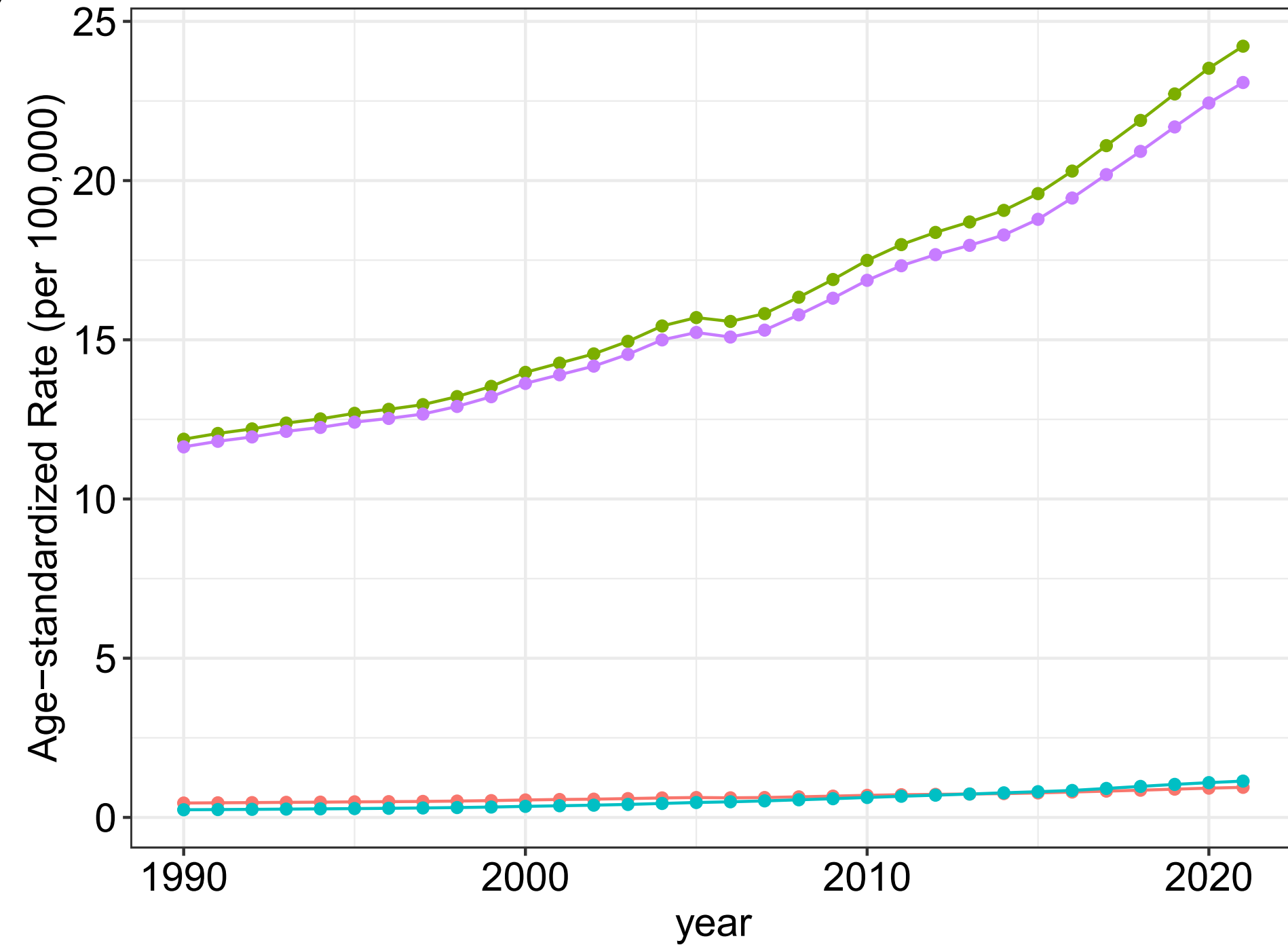

Supplement: Supplementary Figure 2 — Trends in age-standardized rates of deaths, DALYs, YLDs, and YLLs for CRC attributable to high BMI in China and globally from 1990 to 2021. (A) Global age-standardized rates per 100,000 people from 1990 to 2021. (B) China-specific age-standardized rates per 100,000 people during the same period. DALYs, disability-adjusted life years; YLDs, years lived with disability; YLLs, years of life lost; CRC, colorectal cancer; BMI, body mass index. [file DataSheet2.pdf]

**A**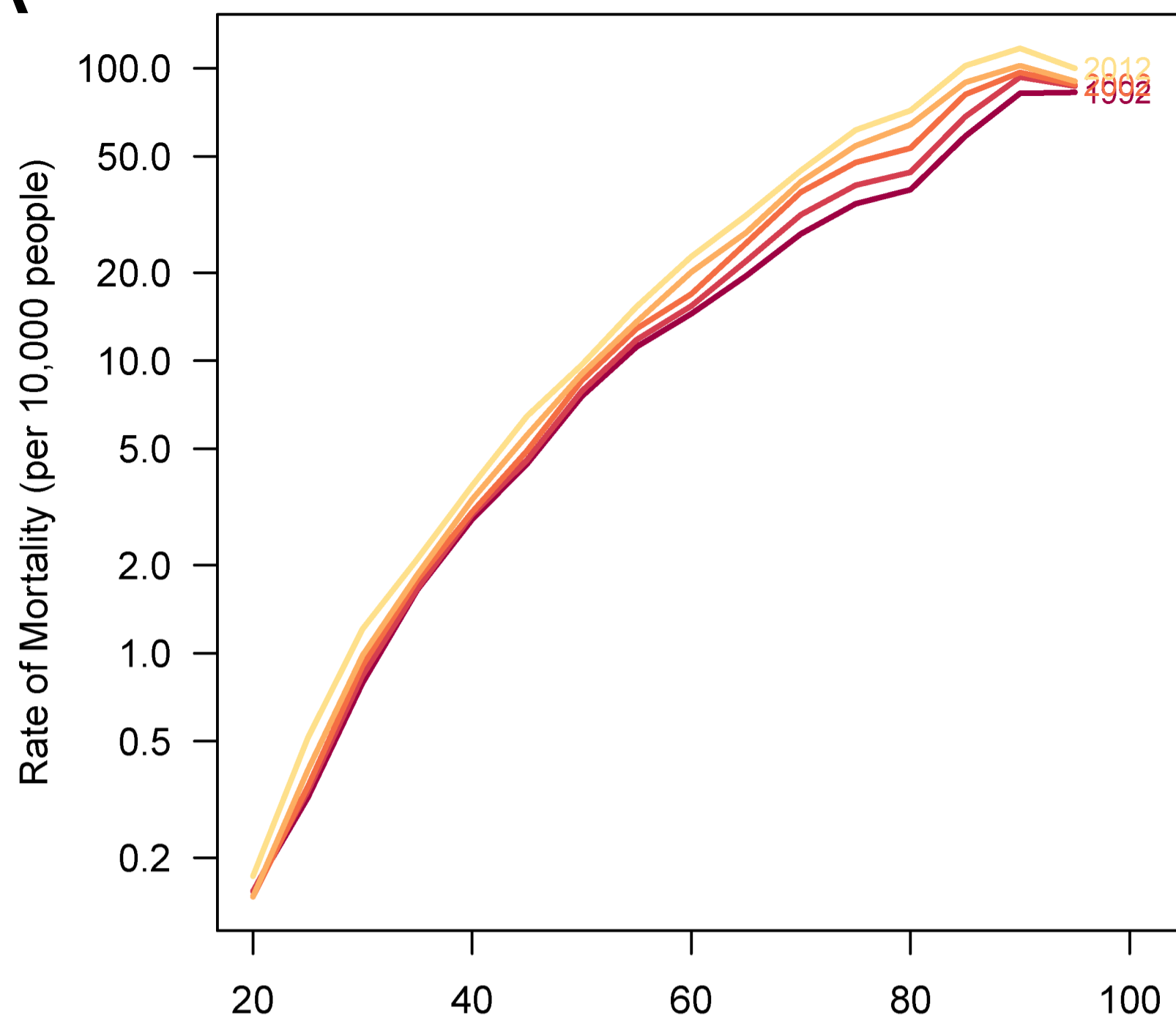**B**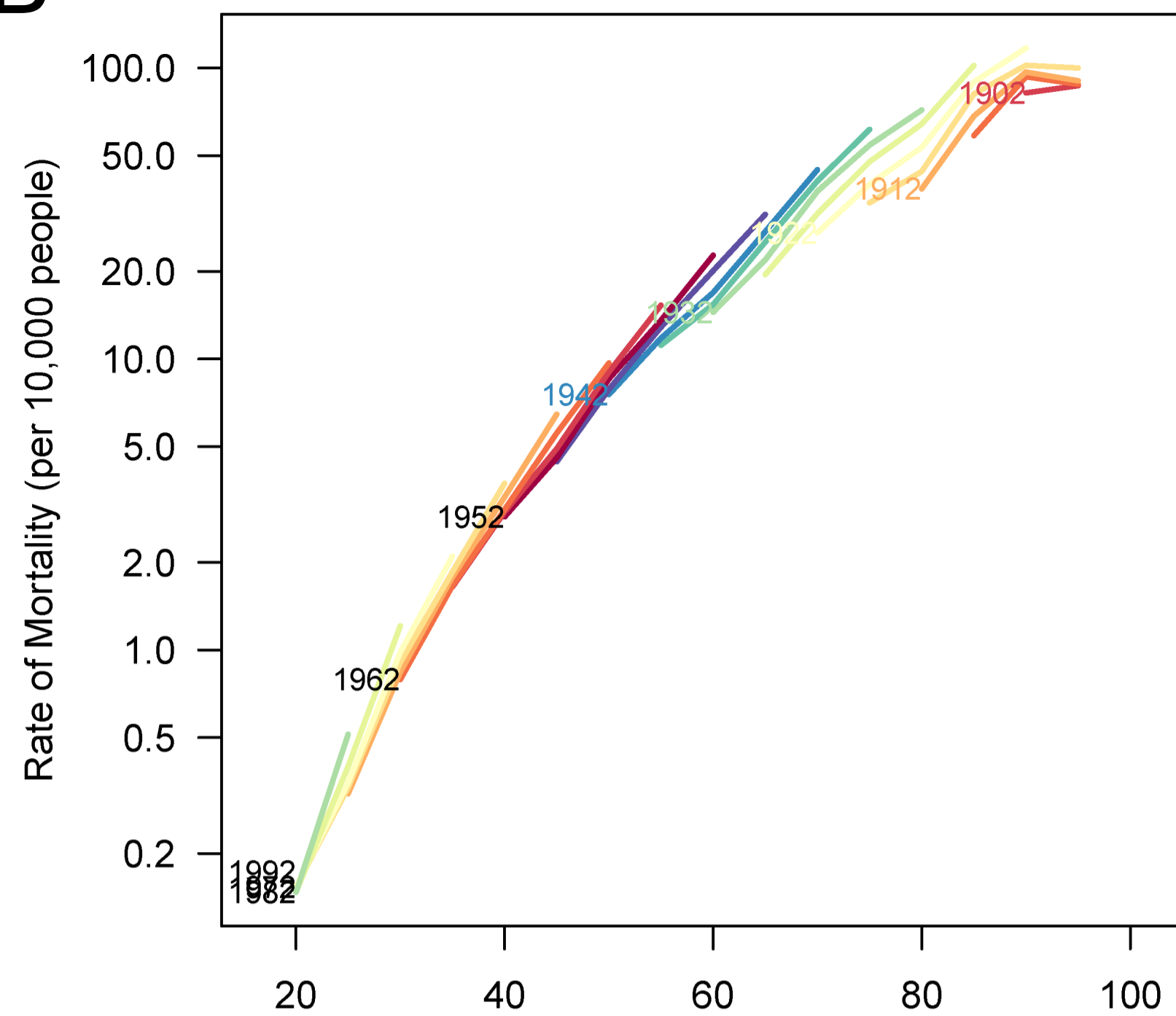**C**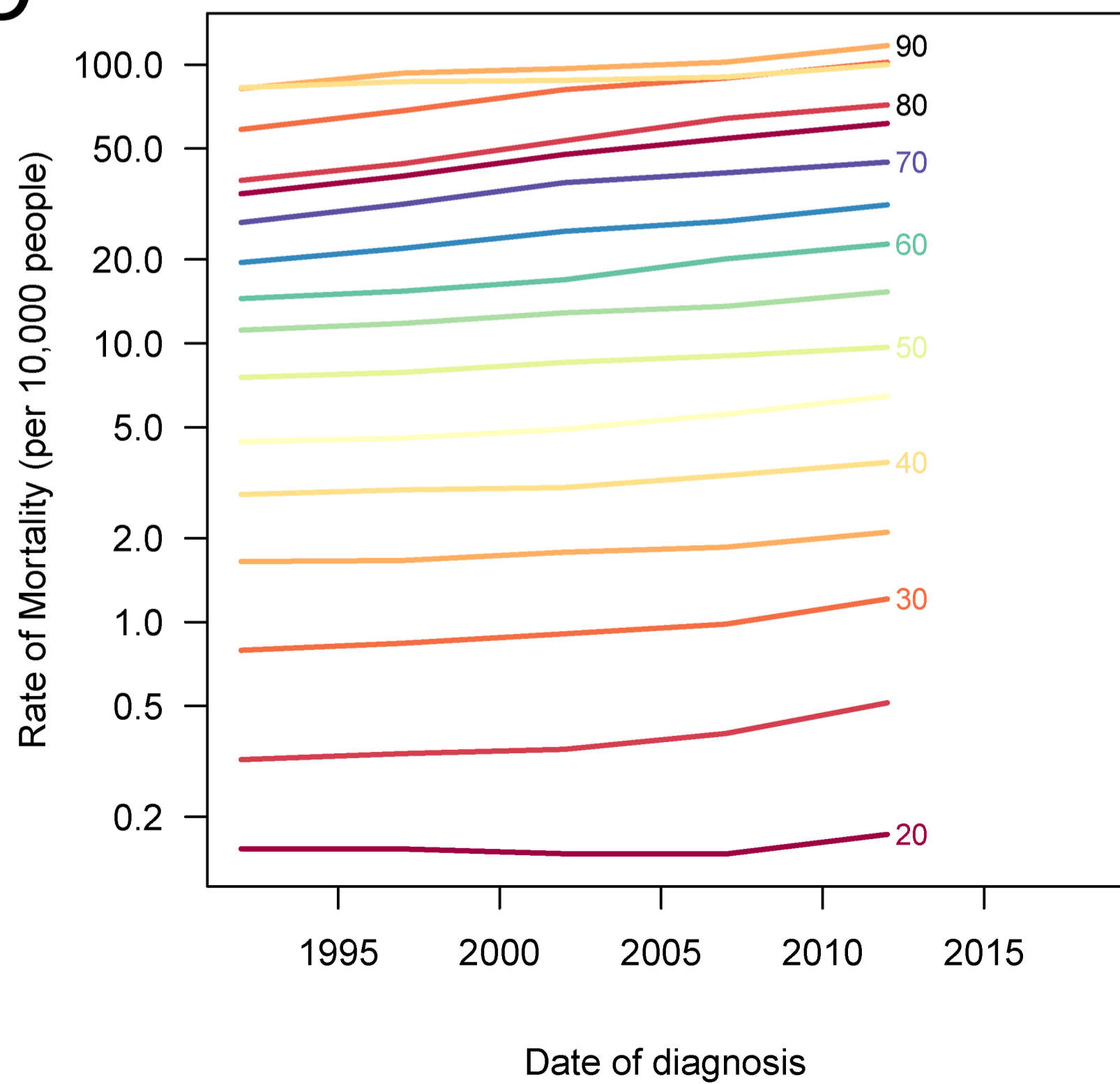**D**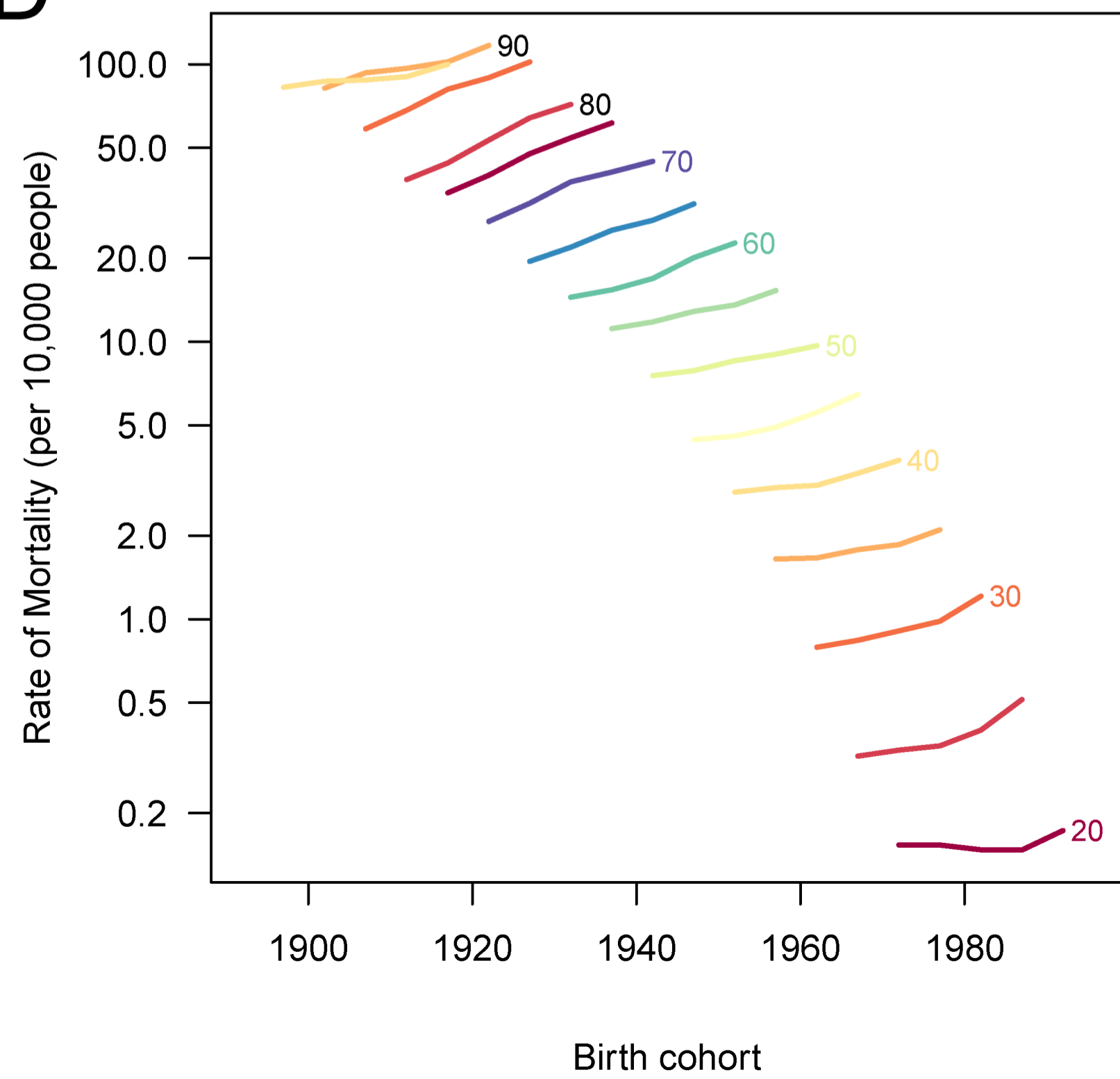

Supplement: Supplementary Figure 3 — Age-period-cohort (APC) analysis of deaths for CRC attributable to high BMI in China. (A) The age-specific mortality rates of CRC across different time periods; each line connects the age-specific mortality rates for a 5-year period, showing an increasing trend with age. (B) The cohort-specific mortality rates of CRC according to birth cohorts; each line connects the cohort-specific mortality rates for a 5-year cohort, indicating a higher risk in more recent cohorts. (C) The period-specific mortality rates of CRC across different age groups; each line connects the period-specific mortality rates for a 5-year age group. (D) The birth cohort-specific mortality rates of CRC across age groups; each line connects the birth cohort-specific mortality rates for a 5-year age group, revealing cohort-based variations in mortality risk. CRC, colorectal cancer; BMI, body mass index. [file DataSheet3.pdf]
