# Supplementary material for: Rising colorectal cancer burden attributable to high body mass index in China from 1990 to 2021: a comprehensive analysis using the global burden of disease study
Source: Front Endocrinol (Lausanne). 2025 May 15;16:1509497. doi: 10.3389/fendo.2025.1509497 (PMC12119292; doi:10.3389/fendo.2025.1509497)
Supplement: Supplementary file 4 [file Table1.docx]

Supplementary Table 1 List of ICD codes mapped to the GBD cause list for causes of death for CRC.

| ICCC3 | ICD 10 | ICD 9 |
| --- | --- | --- |
| XIf2, XIf3 | C18, C18.0, C18.1, C18.2, C18.3, C18.4, C18.5, C18.6, C18.7, C18.8, C18.9, C19, C19.0, C19.9, C2, C20, C20.0, C20.8, C20.9, C21, C21.0, C21.1, C21.2, C21.8, C21.9 | 153, 153.0, 153.1, 153.2, 153.3, 153.4, 153.5, 153.6, 153.7, 153.8, 153.9, 154, 154.0, 154.1, 154.2, 154.3, 154.4, 154.8, 154.9, 209.1, 209.10, 209.11, 209.12, 209.13, 209.14, 209.15, 209.16, 209.17, 569.0, 569.43, 569.44, 569.84, 569.85 |

ICD, International Classification of Diseases; GBD, Global Burden of Disease; CRC, Colon and rectum cancer.
