# Supplementary material for: Rising colorectal cancer burden attributable to high body mass index in China from 1990 to 2021: a comprehensive analysis using the global burden of disease study
Source: Front Endocrinol (Lausanne). 2025 May 15;16:1509497. doi: 10.3389/fendo.2025.1509497 (PMC12119292; doi:10.3389/fendo.2025.1509497)
Supplement: Supplementary file 5 [file Table2.docx]

Supplementary Table 2. Trends in age-standardized mortality, DALY, YLD, and YLL rates (per 100,000 persons) among both sexes, males, and females from 1990 to 2021 for CRC attributable to high BMI in China.

|  | Age-standardized mortality rate | | | Age-standardized DALY rate | | | Age-standardized YLD rate | | | Age-standardized YLL rate | | |
| --- | --- | --- | --- | --- | --- | --- | --- | --- | --- | --- | --- | --- |
| Gender | Period | APC (95% CI) | AAPC (95% CI) | Period | APC (95% CI) | AAPC (95% CI) | Period | APC (95% CI) | AAPC (95% CI) | Period | APC (95% CI) | AAPC (95% CI) |
| Both | 1990-1998 | 1.61 (1.50 - 1.72) ^*^ | 2.43 (2.29 - 2.57) ^*^ | 1990-1998 | 1.30 (1.21 - 1.39) ^*^ | 2.33 (2.21 - 2.45) ^*^ | 1990-1997 | 3.00 (2.79 - 3.21) ^*^ | 5.17 (4.98 - 5.37) ^*^ | 1990-1998 | 1.26 (1.17 - 1.35) ^*^ | 2.24 (2.12 - 2.36) ^*^ |
|  | 1998-2004 | 3.03 (2.82 - 3.24) ^*^ |  | 1998-2004 | 2.64 (2.46 - 2.83) ^*^ |  | 1997-2002 | 5.56 (5.08 - 6.04) ^*^ |  | 1998-2004 | 2.55 (2.38 - 2.73) ^*^ |  |
|  | 2004-2007 | 0.65 (-0.26 - 1.58) |  | 2004-2007 | 0.84 (0.04 - 1.65) ^*^ |  | 2002-2011 | 6.24 (6.07 - 6.40) ^*^ |  | 2004-2007 | 0.68 (-0.09 - 1.46) |  |
|  | 2007-2011 | 3.41 (2.96 - 3.87) ^*^ |  | 2007-2011 | 3.35 (2.95 - 3.75) ^*^ |  | 2011-2016 | 5.02 (4.56 - 5.49) ^*^ |  | 2007-2011 | 3.24 (2.86 - 3.63) ^*^ |  |
|  | 2011-2014 | 1.41 (0.52 - 2.31) ^*^ |  | 2011-2014 | 1.73 (0.95 - 2.52) ^*^ |  | 2016-2019 | 6.99 (5.49 - 8.52) ^*^ |  | 2011-2014 | 1.61 (0.85 - 2.38) ^*^ |  |
|  | 2014-2021 | 3.50 (3.38 - 3.62) ^*^ |  | 2014-2021 | 3.58 (3.48 - 3.69) ^*^ |  | 2019-2021 | 4.81 (3.32 - 6.31) ^*^ |  | 2014-2021 | 3.47 (3.37 - 3.58) ^*^ |  |
| Female | 1990-1997 | 1.32 (1.12 - 1.52) | 1.85 (1.64 - 2.06) ^*^ | 1990-1997 | 1.02 (0.83 - 1.21) ^*^ | 1.63 (1.44 - 1.82) ^*^ | 1990-1997 | 2.96 (2.78 - 3.14) ^*^ | 4.57 (4.38 - 4.76) ^*^ | 1990-1997 | 0.98 (0.80 - 1.16) ^*^ | 1.53 (1.34 - 1.72) ^*^ |
|  | 1997-2004 | 1.94 (1.70 - 2.18) |  | 1997-2004 | 1.43 (1.20 - 1.66) ^*^ |  | 1997-2003 | 4.73 (4.44 - 5.03) ^*^ |  | 1997-2004 | 1.34 (1.12 - 1.56) ^*^ |  |
|  | 2004-2007 | -0.04 (-1.39 - 1.33) |  | 2004-2007 | -0.13 (-1.38 - 1.13) |  | 2003-2006 | 5.64 (4.36 - 6.93) ^*^ |  | 2004-2007 | -0.31 (-1.53 - 0.93) |  |
|  | 2007-2011 | 1.82 (1.14 - 2.50) |  | 2007-2011 | 1.55 (0.93 - 2.18) ^*^ |  | 2006-2016 | 4.45 (4.33 - 4.56) ^*^ |  | 2007-2011 | 1.44 (0.83 - 2.06) ^*^ |  |
|  | 2011-2014 | 0.41 (-0.96 - 1.80) |  | 2011-2014 | 0.65 (-0.63 - 1.94) |  | 2016-2019 | 7.51 (6.20 - 8.84) ^*^ |  | 2011-2014 | 0.52 (-0.74 - 1.79) |  |
|  | 2014-2021 | 3.77 (3.57 - 3.96) |  | 2014-2021 | 3.70 (3.52 - 3.88) ^*^ |  | 2019-2021 | 4.45 (3.15 - 5.76) ^*^ |  | 2014-2021 | 3.58 (3.40 - 3.76) ^*^ |  |
| Male | 1990-1998 | 1.84 (1.69 - 1.99) ^*^ | 2.90 (2.71 - 3.09) ^*^ | 1990-1998 | 1.52 (1.42 - 1.63) | 2.87 (2.75 - 3.00) ^*^ | 1990-1996 | 2.97 (2.54 - 3.40) ^*^ | 5.64 (5.39 - 5.90) ^*^ | 1990-1998 | 1.49 (1.38 - 1.59) ^*^ | 2.78 (2.66 - 2.91) ^*^ |
|  | 1998-2004 | 4.06 (3.77 - 4.35) ^*^ |  | 1998-2004 | 3.68 (3.47 - 3.89) |  | 1996-1999 | 4.74 (2.31 - 7.23) ^*^ |  | 1998-2004 | 3.59 (3.38 - 3.80) ^*^ |  |
|  | 2004-2007 | 1.25 (0.03 - 2.48) ^*^ |  | 2004-2007 | 1.59 (0.69 - 2.49) |  | 1999-2012 | 7.04 (6.90 - 7.19) ^*^ |  | 2004-2007 | 1.44 (0.55 - 2.34) ^*^ |  |
|  | 2007-2011 | 4.48 (3.85 - 5.12) ^*^ |  | 2007-2011 | 4.55 (4.09 - 5.02) |  | 2012-2021 | 5.74 (5.52 - 5.97) ^*^ |  | 2007-2011 | 4.44 (3.99 - 4.90) ^*^ |  |
|  | 2011-2014 | 2.12 (0.89 - 3.36) ^*^ |  | 2011-2015 | 2.63 (2.20 - 3.07) |  |  |  |  | 2011-2015 | 2.52 (2.09 - 2.96) ^*^ |  |
|  | 2014-2021 | 3.29 (3.12 - 3.46) ^*^ |  | 2015-2021 | 3.58 (3.42 - 3.74) |  |  |  |  | 2015-2021 | 3.47 (3.31 - 3.63) ^*^ |  |

AAPC, average annual percent change presented for full period; APC, annual percent change; CI, confidence interval. CRC, colorectal cancer; BMI, body mass index; ^*^, *P*<0.05.
